# Risk of mortality in COVID-19 patients: a meta- and network analysis

**DOI:** 10.1038/s41598-023-29364-8

**Published:** 2023-02-06

**Authors:** Rasoul Kowsar, Amir Mohammad Rahimi, Magdalena Sroka, Alireza Mansouri, Khaled Sadeghi, Elham Bonakdar, Sayed Farshad Kateb, Amir Hossein Mahdavi

**Affiliations:** 1 Department of Animal Sciences, College of Agriculture, Isfahan University of Technology, Isfahan, 84156–83111 Iran; 2 Department of Developmental Biology, Göttingen Center for Molecular Biosciences (GZMB), Georg-August-University, 37073 Göttingen, Germany; 3 University Medical Center Göttingen, Georg-August-University, 37075 Göttingen, Germany; 4 Global Agromedicine Research Center (GAMRC), Obihiro University of Agriculture and Veterinary Medicine, Obihiro, Hokkaido Japan; 5 Yektadam Persian Co., Isfahan, Iran

**Keywords:** Health policy, Viral infection

## Abstract

Understanding the most relevant hematological/biochemical characteristics, pre-existing health conditions and complications in survivors and non-survivor will aid in predicting COVID-19 patient mortality, as well as intensive care unit (ICU) referral and death. A literature review was conducted for COVID-19 mortality in PubMed, Scopus, and various preprint servers (bioRxiv, medRxiv and SSRN), with 97 observational studies and preprints, consisting of survivor and non-survivor sub-populations. This meta/network analysis comprised 19,014 COVID-19 patients, consisting of 14,359 survivors and 4655 non-survivors. Meta and network analyses were performed using META-MAR V2.7.0 and PAST software. The study revealed that non-survivors of COVID-19 had elevated levels of gamma-glutamyl transferase and creatinine, as well as a higher number of neutrophils. Non-survivors had fewer lymphocytes and platelets, as well as lower hemoglobin and albumin concentrations. Age, hypertension, and cerebrovascular disease were shown to be the most influential risk factors among non-survivors. The most common complication among non-survivors was heart failure, followed by septic shock and respiratory failure. Platelet counts, creatinine, aspartate aminotransferase, albumin, and blood urea nitrogen levels were all linked to ICU admission. Hemoglobin levels preferred non-ICU patients. Lower levels of hemoglobin, lymphocytes, and albumin were associated with increased mortality in ICU patients. This meta-analysis showed that inexpensive and fast biochemical and hematological tests, as well as pre-existing conditions and complications, can be used to estimate the risk of mortality in COVID-19 patients.

## Introduction

Globally, healthcare workers encounter challenges in reducing coronavirus disease of 2019 (COVID‐19) severity and mortality^1^. Many subpopulations of patients with mild to non-severe COVID‐19 experience serious problems or even death, which is a growing concern^1,2^. According to reports, approximately 19% of COVID-19 patients experienced serious illness and 61.5% died within 28 days of admission, while 50% of hospitalized patients had no significant clinical and medical remission after 10 days^3–5^. Therefore, early diagnosis of patients with a possible serious COVID-19 infection and a high risk of mortality will relieve pressure on medical services, since treating a large number of patients places a significant burden on medical resources. The role of risk prediction is drastically shifting, and it helps to effectively determine if preventive protocols and treatment of positive cases are attempted. Therefore, early prognosis and care for this patient group are crucial to limiting disease progression and death^1^. In general, hematological predictors, risk factors, and potential complications of COVID-19 mortality and intensive care unit (ICU) referral need further investigation. Understanding the relevance of each risk factor to disease progression and mortality can assist in recognizing at-risk subpopulations and evaluating healthcare quality. Efforts should also be made to prepare for risk groups and estimate the risk of fatality in order to better understand the true patterns of mortality^6^. For example, age and gender have been identified as well-known risk factors for severe COVID19 outcomes: about 90% of COVID-19-related deaths in the United Kingdom (UK) have occurred in people over the age of 60, with 60% occurring in men^7^. Pre-existing conditions (such as tobacco use, coronary disease, hypertension, diabetes, respiratory and renal disease, and cancer) have also been related to an elevated risk of death^6^. For example, the Chinese Center for Disease Control and Prevention reported in a survey of 44,672 people (1023 deaths) that cardiovascular diseases, hypertension, diabetes, respiratory diseases, and cancers were associated with a high risk of death from COVID-19^8^.

The aim of this meta-analysis was to assess the relevance of hematological/biochemical indices, pre-existing conditions, and complications to COVID-19 mortality and ICU referral and death, assuming an association between these factors. Furthermore, the association between hematological/biochemical indices, pre-existing conditions, and complications in COVID-19 non-survivors was investigated. The primary outcomes of this study were COVID-19 mortality and survival, and exposures included pre-existing conditions (such as age, gender, smoking, alcohol consumption, and so on) and comorbidities (i.e., hypertension, cerebrovascular diseases, diabetes, any comorbidities, cardiovascular diseases, renal diseases, chronic obstructive pulmonary disease (COPD), cancer, and liver diseases).

A network analysis was also used to estimate complex patterns of interaction^9^, to assess the specific structure of interrelationships between various factors, and to classify the degree of centrality and connectivity of patient characteristics/prognostic features to COVID-19 mortality.

## Methods

### Assessment of multicollinearity and correlation in influencing factors

The variance inflation factor (VIF) was used to determine whether any comorbidities or hematological and biochemical indices contribute to complications in COVID-19 patients. Indeed, VIF is used to calculate the amount of multicollinearity in variables, i.e., influencing factors. As a rough rule of thumb, VIF > 1 means that the variables are subject to collinearity, and VIF > 10 means that the correlation (collinearity) between the variables is very high. In addition, bivariate Pearson and Spearman correlations were used to assess linearity or nonlinearity between influencing factors (i.e., blood index and comorbidities) and complications. Correlation is significant at the 0.05 level (Bonferroni-corrected P value). Since the Anderson–Darling test confirmed that the data were normal, the Pearson correlation was used for network analysis.

### Search strategy

A literature review was performed between December 2019 and April 2020 (no language restrictions were applied) in PubMed, Scopus, and various preprint servers (bioRxiv, medRxiv and SSRN) using MeSH keywords/terms, such as “COVID-19” AND “novel coronavirus” AND “new coronavirus” AND “coronavirus-2019” AND “COVID-2019” AND “SARS-COV-2” AND “2019-nCOV”.

### Data extraction and analysis

The results of the search strategy were initially evaluated using abstracts and titles. Following that, the full text of the related articles was then checked using the inclusion and exclusion criteria. The final list of papers included was contrasted, and the disagreements were settled by a consensus discussion between authors. Three researchers (RK, AM, and AMR) independently extracted data such as the type and date of release, country, sample size, age, sex, blood indices and parameters, pre-existing health conditions, and complications. Using a structured spreadsheet, three authors (RK, AMR, and MS) tested the consistency of the data obtained. The Anderson–Darling test was used to determine if the resulting data was normal. For Meta-analysis and network analysis, META-MAR V2.7.0 and PAST were used, respectively. The Standardized Mean Difference (SMD) was used to identify the effect size of various hematological/biochemical indices, risk factors (pre-existing conditions), and complications in COVID-19 survivors and non-survivors, or in ICU-admitted patients (including both survivors and non-survivors) and non-ICU patients. Due to heterogeneity, random-effect models were used to calculate the weighted mean effect sizes and 95% confidence interval (CI).

### Inclusion criteria

**Figure 1:**
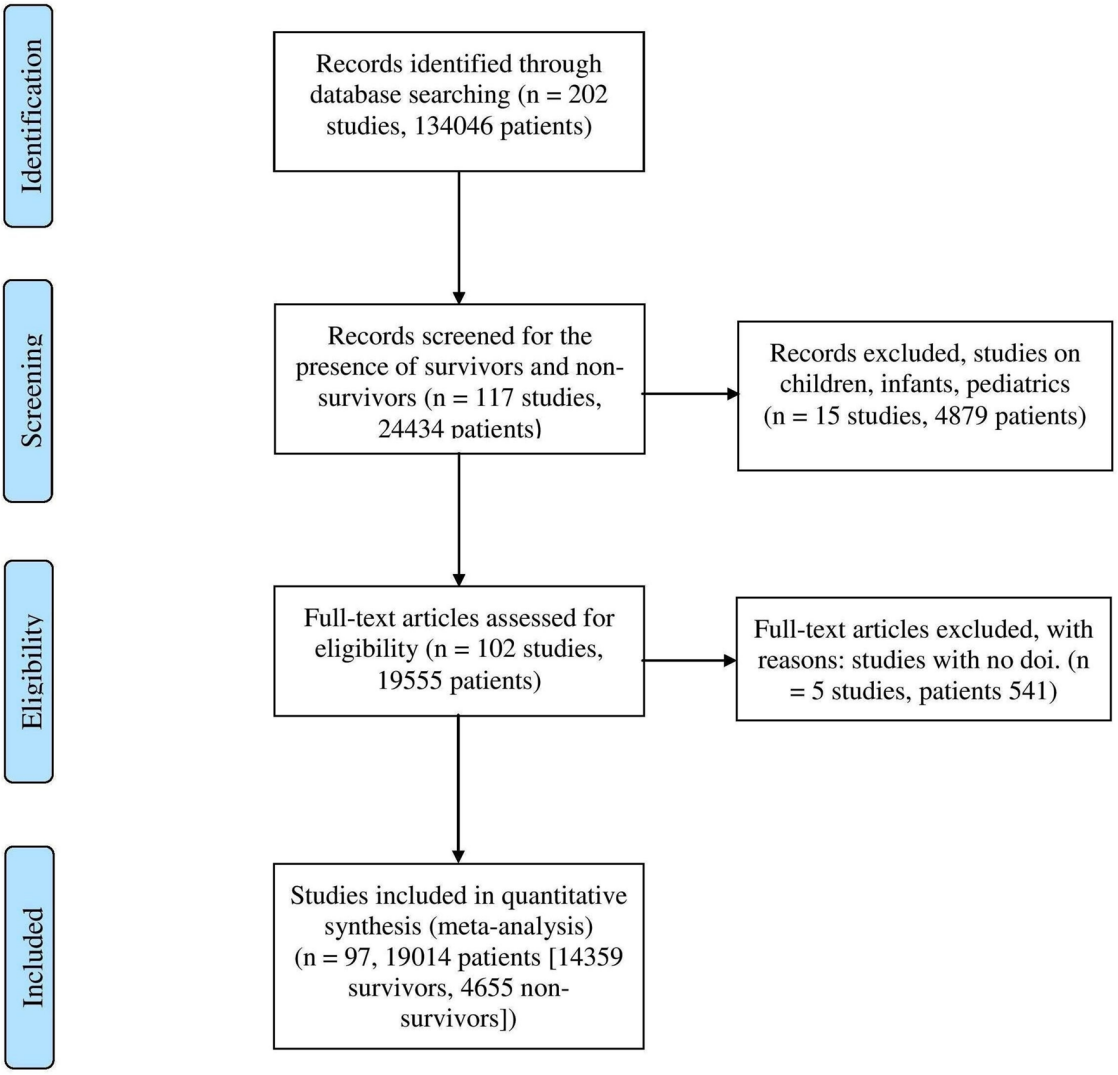
PRISMA flow diagram of the studies identified, screened, reviewed, and included in the meta-analysis.

Figure 1 shows the flow diagram of studies assessed for inclusion. We collected data from research that recorded, at least, one of the following criteria, such as hematological/biochemical indices, pre-existing conditions, demographic factors, complications, and clinical outcomes (i.e., mortality or survival).

Pre-existing health conditions were described as conditions that existed prior to the patient’s arrival at the emergency department/hospital, and the decision was based on the medical record^10^. Complications (i.e., heart failure, septic shock, acidosis, respiratory failure, coagulopathy, acute renal injury, liver dysfunction, and secondary infection) were unanticipated events, diseases or symptoms triggered by the combination of a given disease^11^ and pre-existing conditions. Importantly, complications can occur even though appropriate precautions are taken. COVID-19 has been found to cause serious medical complications and death, especially in elderly patients and people with pre-existing health conditions.

### Exclusion criteria

Data from over 200 reported clinical studies and preprints were screened. Following a comprehensive review of the data in figures and tables, 105 reports were excluded due to a lack of survivor or non-survivor sup-groups, examining infants/children/pediatrics, or having no DOI. This resulted in 97 eligible observational studies (19,014 patients)^4,12–107^, with survivors (n = 14,359) and non-survivors (n = 4655) as strict subpopulations.

The reference list of papers and other systematic reviews were also scrutinized. The hematological and biochemical indices were checked to ensure they were the same, unless otherwise converted to the same unit. In terms of inclusion and exclusion criteria, paper selection resulted in different sample sizes for different parameters between survivors and non-survivors. These studies included patients from China, Italy, Scotland, the United States, UK, Japan, Singapore, South Korea, Iceland, Chile, the Netherlands, and Germany. Supplementary Table 1 describes the characteristics of the studies selected for meta-analysis.

### Heterogeneity and risk of bias in individual studies

The I^2^ and Tau^2^ statistics were used to determine statistical heterogeneity^108^. According to the Cochrane's handbook for Systematic Reviews of Interventions, the I^2^ expresses the proportion of variance due to heterogeneity (i.e., 30% to 60%, 50% to 90% and 75% to 100% correspond to moderate, substantial and considerable degrees of heterogeneity, respectively)^109^. In addition, a meta-regression analysis was performed to assess the effect of variables on the effect size. The Z-test and its associated P-values determined whether the observed prevalence varied from zero percent.

Moreover, the Quality in Prognostic Studies (QUIPS) tool, a non-validated instrument with space for personal interpretation, was employed to assess the risk of bias in the studies^110,111^. The Cochrane Methods Prognosis group recommends the QUIPS tool for prognosis research because it tackles all common sources of bias^110,111^. Based on this, we concluded that the QUIPS method was sufficient for determining bias risk. Two team members (AM and RK) independently assessed the risk of bias in each study and classified it as low, moderate or high risk^112^. Any disagreements were resolved by consensus. The QUIPS tool includes domains on: study participation, study attrition, prognostic factor measurement, outcomes measurement, statistical analysis and reporting, and study confounding^113^.

Studies with a low risk of bias had thorough explanations of the population, design, and measures; as well as clear descriptions of how the measure was executed, equipments used, and how the data were interpreted. In studies with a medium risk of bias, there was some bias, but not enough to invalidate the data. These studies do not meet all of the requirements for a low risk of bias ranking, but no error is likely to result in significant bias. Studies with a high risk of bias had major flaws, indicating different forms of bias that might invalidate the findings. The high-risk study included one or more crucial or “fatal” flaws in its design, analysis, or reporting, as well as significant amounts of missing information^112^ The findings of the risk of bias assessment were identified in the narrative synthesis but were not included in the meta-analysis.

### Network analysis

**Figure 2:**
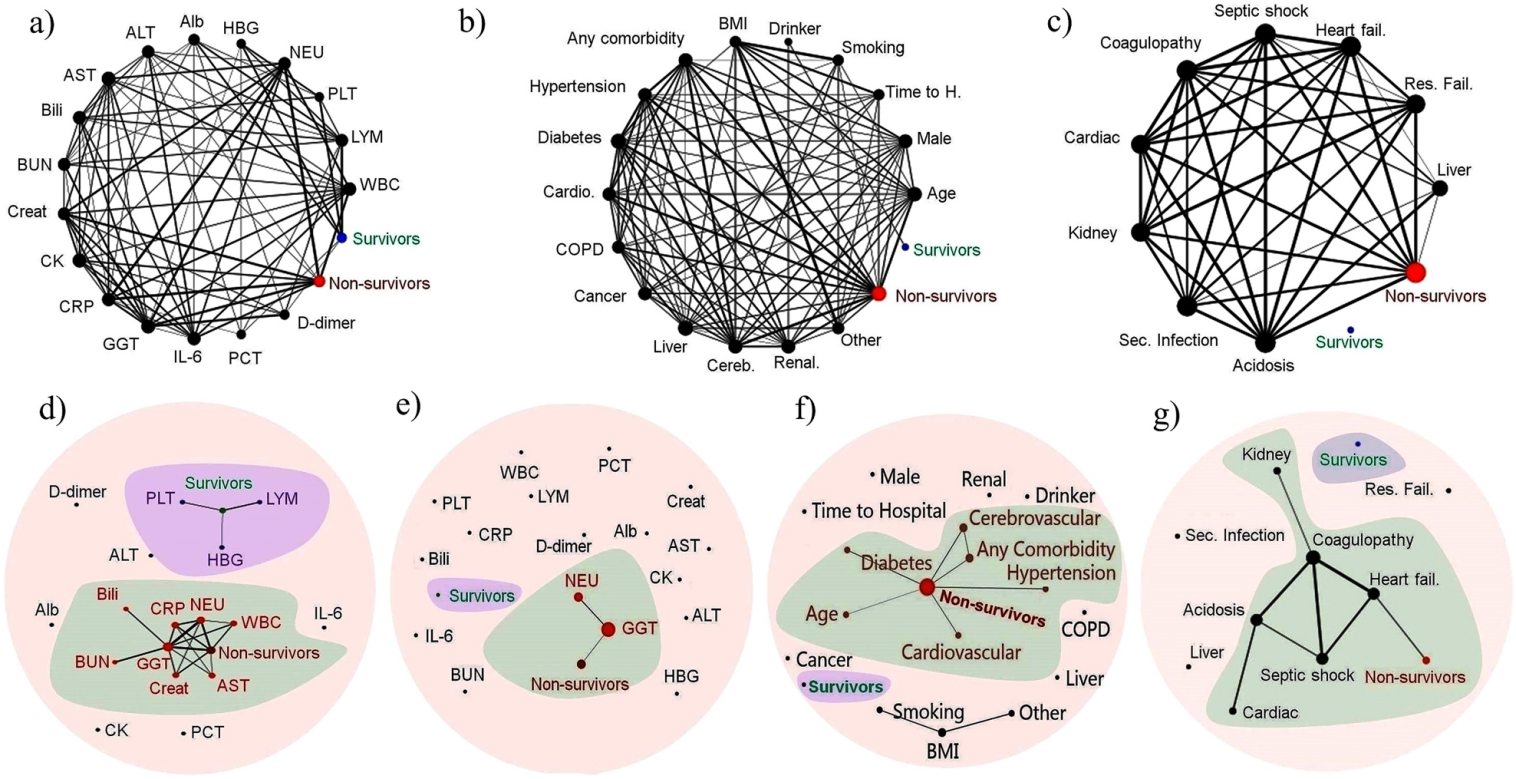
Correlation-based network analysis. The Pearson correlation threshold of 50% was used to show the network of all variables (**a**–**c**). More precisely, the Pearson correlation thresholds of 72% and 93% (**d**, **e**) were respectively selected to define the connection between survivors, non-survivors and blood parameters. The Pearson correlation thresholds of 79% and 97% were respectively chosen to assess the relationship between survivors, non-survivors and (**f**) risk factors or (**g**) complications. Circles of the network indicate the blood parameters (**a**, **d**, **e**), risk factors (**b**, **f**) and complications (**c**, **g**). The size of the node reflects the degree of connectivity of the node and the edges display the relationship between the two variables. The thicker edges reveal higher correlations between variables. Nodes with more links are close to each other. Network analysis and visualization was carried out using PAST and Fruchterman-Reingold algorithm or Circular algorithm as a force-directed layout algorithm. Abbreviations in panels (**a**), (**d**), and (**e**): Alb, albumin; HBG, hemoglobin; NEU, neutrophil; PLT, platelet; LYM, lymphocyte; WBC, white blood cells, PCT, procalcitonin; GGT, gamma-glutamyl transferase; CRP, C-reactive protein, CK, creatine kinase; Creat: creatinine, BUN, blood urea nitrogen; Bili, total bilirubin; AST, aspartate aminotransferase; ALT, alanine aminotransferase. Abbreviations in panels (**b**) and (**f**): BMI, body mass index; Time to H, time from symptoms appearance to hospitalization; Renal, renal disease; Cereb, cerebrovascular disease; Liver, liver disease; COPD, chronic obstructive pulmonary disease; Cardio, cardiovascular disease. Abbreviations in panels (**c**) and (**g**): Fail, failure; Res, respiratory; Liver, liver dysfunction; Sec, secondary; Kidney, acute kidney injury; Cardiac, acute kidney injury.

Data from variable, such as blood indices, pre-existing conditions (i.e., male, liver diseases, renal diseases, cerebrovascular diseases, diabetes, COPD, drinking, smoking, any comorbidities, cancer, cardiovascular diseases, hypertension), or complications (i.e., heart failure, respiratory failure, secondary infection, coagulopathy, acidosis, liver dysfunction, septic shock, acute cardiac injury, and acute kidney injury) were used for network analysis as the proportion of each parameter obtained from each study. In terms of parameters, such as age, time to hospital and BMI, the mean of each parameter obtained from each study was used for network analysis. Then data were classified in a binary manner for network analysis, with 0 and 1 representing mortality and survival, respectively. The PAST software (accessible at: http://folk.uio.no/ohammer/past) was used to implement the Circular and Fruchterman-Reingold algorithms as a force-directed layout algorithm. The Anderson–Darling test confirmed the normality of data, so the Pearson similarity index was used as a parametric index for network analysis. The Pearson correlation threshold of 50% (as a basic level) was used to establish the network of all variables. To be more specific, Pearson correlation thresholds of 72% and 93% were respectively selected to characterize the relationship between blood indices and outcomes (i.e., survivors or non-survivors, Fig. 2a–g). Pearson correlation thresholds of 79% and 97% were selected to determine the relationship between risk factors (pre-existing conditions) or complications and outcomes (i.e., survivors or non-survivors, Fig. 2f,g). These cutoff points were selected because there was no subsequent interaction among the variables after these thresholds. The node’s size represents its degree of connectivity, and the edges display the relationship between the two variables. The thicker edges indicate stronger correlations between variables. Nodes with more links are closer together. Small nodes and thin edges represent small values.

## Results

### Data characteristics and multicollinearity among the influencing factors

Table 1 presents the clinical outcomes, and Appendix 1 describes the data collection process for Table 1. The patient’s age (n = 9375) ranged from 25.3 to 80.0 years (49.8; CI_95%_ [46.9, 52.7]). There were 5448 survivors (age: 46.6; CI_95%_ [44.2, 48.9]) and 3927 non-survivors (age: 71.5; CI_95%_ [66.4, 76.5]). Males outnumbered females in the non-survivor population (33.3 vs. 17.7%).

**Table 1:** COVID-19 patient characteristics, blood parameters, pre-existing conditions, and complications**.**

Characteristic	All Patients	Survivors	Non-survivors
Age—no, yr, mean (confidence interval, CI_95%_)	9375, 49.8 (46.9, 52.7)	5448, 46.6 (44.2, 48.9)	3927, 71.5 (66.4, 76.5)
Gender			
Male—no./total no. (%)	9599/17,778 (54.0)	6399/9599 (66.7)	3200/9599 (33.3)
Female—no./total no. (%)	8179/17,778 (46.0)	6733/8179 (82.3)	1446/8179 (17.7)
Drinker			
Yes—no./total no. (%)	253/1606 (15.8)	243/253 (96.0)	10/253 (2.8)
No—no./total no. (%)	1353/1606 (84.2)	1262/1353 (93.3)	91/1353 (6.7)
Smoker			
Yes—no./total no. (%)	874/7583 (11.5)	843/874 (96.5)	31/874 (3.5%)
No—no./total no. (%)	6709/7583 (88.5)	6377/6709 (95.1)	332/6709 (4.9)
Days to Hospital—no, mean (CI_95%_)	5456, 5.9 (5.8, 6.0)	2109, 5.7 (5.5, 5.8)	3347, 8.0 (7.9, 8.1)
Body mass index—no, mean (CI_95%_)	383, 24.5 (24.3, 24.7)	314, 24.1(24.0, 24.3)	69, 25.5 (24.7, 26.3)
Any comorbidities			
Yes—no./total no. (%)	6321/11,473 (55.1)	2156/6321 (34.1)	4165/6321 (65.9)
No—no./total no. (%)	5152/11,473 (44.9)	4873/5152 (94.6)	279/5152 (5.4)
Hypertension			
Yes—no./total no. (%)	4443/14,080 (31.6)	1579/4443 (35.5)	2864/4443 (64.5)
No—no./total no. (%)	9637/14,080 (68.4)	8256/9637 (85.7)	1381/9637 (14.3)
Diabetes			
Yes—no./total no. (%)	2222/14,423 (15.4)	766/2222 (34.5)	1456/2222 (65.5)
No—no./total no. (%)	12,201/14,423 (84.6)	9111/12,201 (74.7)	3090/12,201 (25.3)
Cardiovascular disease			
Yes—no./total no. (%)	1679/14,189 (11.8)	355/1679 (21.1)	1323/1679 (78.8)
No—no./total no. (%)	12,510/14,189 (88.2)	9202/12,510 (73.6)	3308/12,510 (26.4)
Chronic obstructive lung (COPD)			
Yes—no./total no. (%)	843/11,925 (7.1)	218/843 (25.9)	625/843 (74.1)
No—no./total no. (%)	11,082/11,925 (92.9)	7408/11,082 (66.8)	3674/11,082 (33.2)
Cancer			
Yes—no./total no. (%)	952/11,602 (8.2)	191/952 (20.1)	761/952 (79.9)
No—no./total no. (%)	10,650/11,602 (91.8)	6965/10,650 (65.4)	3685/10,650 (34.6)
Liver disease			
Yes—no./total no. (%)	293/7783 (3.8)	121/293 (41.3)	172/293 (58.7)
No—no./total no. (%)	7490/7783 (96.2)	3806/7490 (50.8)	3684/7490 (49.2)
Cerebrovascular disease			
Yes—no./total no. (%)	233/4172 (5.6)	121/233 (51.9)	112/233 (48.1)
No—no./total no. (%)	3939/4172 (94.4)	3368/3939 (85.5)	571/3939 (14.5)
Renal disease			
Yes—no./total no. (%)	797/11,551 (6.9)	91/797 (11.4)	706/797 (88.6)
No—no./total no. (%)	10,754/11,551 (93.1)	7546/10,754 (70.2)	3209/10,754 (29.8)
Other disease—no./total no. (%)	682/10,562 (6.5)	437/682 (64.1)	245/690 (35.9)
Severity			
Mild—no./total no. (%)	4041/19,014 (21.2)	4041/4041 (100.0)	0/4041 (0.0)
Severe—no./total no. (%)	4874/19,014 (25.6)	498/4874 (10.2)	4376/4874, 89.8
Unreported—no./total no. (%)	10,099/19,014 (53.2)	9800/10,099 (97.0)	299/10,099 (3.0)
Treatment			
Antibiotics—no./total no. (%)	9542/19,042 (50.1)	5817/9542 (61.0)	3725/9542 (39.0)
Antiviral drugs—no./total no. (%)	10,371/19,042 (54.5)	5353/10,371 (51.6)	5018/10,371 (48.4)
Intensive care unit (ICU) admission			
Non-ICU—no./total no. (%)	4379/11,420 (38.3)	4379/4379 (100.0)	0/4379 (0.0)
ICU-Endpoint—no./total no. (%)	6754/11,420 (59.2)	6223/6754 (92.1)	531/6754 (7.9)
ICU-Only—no./total no. (%)	287/11,420 (2.5)	124/287 (43.2)	163/287 (56.8)
Ethnics			
White, European—no./total no. (%)	4817/18,442 (26.1)	1176/4817 (24.4)	3641/4817 (75.6)
African American—no./total no. (%)	402/18,442 (2.2)	364/402 (90.5)	38/402 (9.5)
Asian—no. (%)	11,632/18,442 (63.1)	10,821/11,632 (93.0)	811/11,632 (7.0)
Hispanic, Latino—no./total no. (%)	922/18,442 (5.0)	922/922 (100.0)	0/922 (0.0)
Multi-ethnicity—no./total no. (%)	669/18,442 (3.6)	665/669 (99.4)	4/669 (0.6)
Hematological indices—n, mean 10^9^/L (CI_95%_)			
White blood cells	7174, 5.95 (5.52, 6.38)	6591, 5.43 (5.17, 5.70)	583, 8.55 (6.74, 10.36)
Lymphocyte	7640, 1.11 (1.01, 1.22)	6943, 1.23 (1.12, 1.34)	697, 0.60 (0.52, 0.68)
Platelet	7262, 179.49 (172.06, 187.08)	6572, 187.76 (181.22, 194.32)	690, 149.92 (132.87, 166.96)
Neutrophil	4841, 4.19 (3.73, 4.65)	4237, 3.52 (3.24, 3.81)	604, 6.48 (5.39, 7.57)
Biochemical indices—n, mean (CI_95%_)			
Hemoglobin—g/L	6379, 132.58 (130.14, 135.01)	5887, 134.44 (131.95, 136.92)	492, 124.03(118.72, 129.34)
Albumin—g/L	3491, 36.75 (33.71, 39.79)	3040, 38.51 (3460, 42.41)	451, 31.93 (30.15, 33.70)
Alanine aminotransferase—U/L	5156, 29.41 (25.22, 33.60)	4705, 29.27 (24.30, 34.23)	548, 30.09 (23.38, 36.80)
Aspartate aminotransferase—U/L	5020, 33.75 (29.17, 38.32)	4526, 30.06 (26.48, 33.65)	494, 50.68 (32.23, 69.13)
Gamma-glutamyl transferase—U/L	1642, 19.35 (5.06, 33.65)	1582, 11.06 (2.69, 19.43)	60, 52.5 (36.88, 68.12)
Total bilirubin—µmol/L	4758, 13.87 (10.94, 16.79)	4206, 13.41 (9.74, 17.08)	552, 15.47 (11.60, 19.34)
Blood urea nitrogen—mmol/L	3801, 6.35 (4.30, 8.40)	3326, 5.55 (2.82, 8.28)	475, 8.55 (7.08, 10.02)
Creatinine—µmol/L	5306, 69.95 (65.86, 74.04)	4670, 64.64 (61.74, 67.53)	636, 87.52 (76.62, 98.41)
Creatine Kinase—U/L	4044, 79.46 (68.26, 90.69)	3706, 73.20 (64.34, 82.06)	338, 101.0 (56.75, 145.25)
C-reactive protein—mg/L	4895, 37.36 (26.03, 48.70)	4297, 22.32 (14.63, 30.00)	598, 96.39 (63.46, 129.31)
Interleukin-6—pg/mL	1827, 31.69 (7.44, 55.93)	1557, 18.96 (− 9.93, 47.86)	270, 59.68 (8.27, 111.09)
Procalcitonin—ng/mL	4012, 0.68 (− 0.30, 1.67)	3374, 0.78 (− 0.58, 2.14)	638, 0.41 (0.19, 0.62)
D-dimer—mg/L	4053, 6.78 (− 0.13, 13.69)	3390, 7.08 (− 2.08, 16.25)	663, 5.81 (3.00, 8.63)
Complications			
Liver dysfunction—no./total no. (%)	602/3037 (19.8)	487/602 (80.9)	115/602 (19.1)
Respiratory failure—no./total no. (%)	3782/6313 (59.9)	236/3782 (6.2)	3546/3782 (93.8)
Heart failure—no./total no. (%)	189/2148 (8.8)	21/189 (11.1)	168/189 (88.9)
Septic shock—no./total no. (%)	178/4567 (3.9)	19/178 (10.7)	159/178 (89.3)
Coagulopathy—no./total no. (%)	51/1287 (4.0)	14/51 (27.5)	37/51 (72.5)
Acute cardiac injury—no./total no. (%)	732/5479 (13.4)	93/732 (12.7)	639/732 (87.3)
Acute kidney injury—no./total no. (%)	1155/6936 (16.7)	63/1155 (5.5)	1092/1155 (94.5)
Secondary infection—no./total no. (%)	617/4993 (12.4)	202/617 (32.7)	415/617 (67.3)
Acidosis—no./total no. (%)	47/515 (9.1)	15/47 (31.9)	32/47 (68.1)

Both Pearson and Spearman analyzes were performed to examine linearity and nonlinearity between variables. Bivariate correlation analysis revealed that certain hematological and biochemical indicators were associated with complications in COVID-19 patients (see Supplementary Table 2).

Liver dysfunction had no linear relationship with blood index, but Spearman analysis showed a significant nonlinear relationship with gamma-glutamyltransferase (GGT) levels (r = 0.80), interleukin (IL)-6 (r = 0.80), c-reactive protein (CRP) levels (r = 0.70), creatinine (Cr) levels (r = 0.70), platelets (PLT) numbers (r = 0.70), alanine aminotransferase (ALT, r = 0.69), and higher white blood cells (WBC) counts (r = 0.68) (see Supplementary Table 2).

Using Pearson analysis, elevated gamma-glutamyltransferase (GGT) levels (r = 0.91) and decreased platelet count (PLT) (r = -0.75) were found to be significantly associated with respiratory failure. Interestingly, Spearman analysis revealed that respiratory failure was strongly associated with all blood indices except LYM, GGT, and albumin (see Supplementary Table 2).

Pearson analysis revealed that heart failure was significantly associated with higher GGT levels (r = 0.88), higher white blood cells (WBC) counts (r = 0.77), higher blood urea nitrogen (BUN) levels (r = 0.89), and lower PLT counts (r = − 0.83). Spearman found no significant non-linear relationship between heart failure and blood indices (see Supplementary Table 2).

Using Pearson analysis, we found that septic shock was significantly associated with higher Cr (r = 0.75) and lower PLT counts (r = − 0.76). However, Spearman analysis showed a positive association between septic shock and IL-6 (r = 0.90), BUN (r = 0.86), and creatine kinase (CK, r = 0.85) (see Supplementary Table 2).

Pearson correlation showed that acute cardiac injury was significantly correlated with higher GGT level (r = 0.75), higher Cr level (r = 0.70), and lower PLT counts (r = − 0.85). Spearman correlation identified an association between acute cardiac injury and BUN (r = 0.87) and CK (r = 0.87). Acute kidney injury was significantly correlated with higher GGT levels using Pearson (r = 0.77) and Spearman (r = 0.89) analysis (see Supplementary Table 2).

Pearson analysis showed that secondary infection was associated with higher neutrophil (NEU) counts (r = 0.76). Spearman analysis showed a trend (*P* = 0.06) to associate secondary infection with IL-6 (r = 0.76) and BUN (r = 0.76) (see Supplementary Table 2).

Additionally, we found associations with certain pre-existing health conditions and complications in COVID-19 patients (see Supplementary Table 3). According to the Pearson and Spearman analysis, there was no significant relationship between liver dysfunction and pre-existing health conditions in COVID-19 patients. Spearman analysis revealed that age (r = 0.77), comorbidities (r = 0.74), and diabetes (r = 0.69) were significantly associated with respiratory failure. Pearson analysis found no significant association between respiratory failure in COVID-19 patients and pre-existing health problems (see Supplementary Table 3).

Pearson analysis showed that age (r = 0.80), comorbidities (r = 0.78), cardiovascular disease (r = 0.79) and cerebrovascular disease (r = 0.75) were all significantly correlated with heart failure in COVID-19 patients. Spearman analysis showed that heart failure in COVID-19 patients was associated with cerebrovascular disease (r = 0.88), comorbidities (r = 0.82), diabetes (r = 0.81), cardiovascular disease (r = 0.71) and renal disease (r = 0.72) (see Supplementary Table 3).

Using Pearson analysis we found that septic shock was associated with cerebrovascular diseases (r = 0.73) in COVID-19 patients. Spearman analysis showed that septic shock was associated with diabetes (r = 0.77), comorbidities (r = 0.73), age (r = 0.70), hypertension (r = 0.70), and cardiovascular disease (r = 0.69) (see Supplementary Table 3).

Pearson analysis found no significant associations between acute cardiac injury, acute kidney injury, or secondary infections and pre-existing health conditions in COVID-19 patients. However, Spearman analysis detected a significant association between acute cardiac injury and COPD (r = 0.78), renal disease (r = 0.72), or cardiovascular disease (r = 0.70). Spearman analysis confirmed a significant association between renal disease (r = 0.70) or cardiovascular disease (r = 0.68) and COVID-19 complications, acute kidney injury. Using Spearman analysis, secondary infections were found to be associated with comorbidities (r = 0.83), cardiovascular disease (r = 0.88), cerebrovascular disease (r = 0.77), and diabetes (r = 0.70) (see Supplementary Table 3).

Furthermore, we found a very high VIF in blood indices as influencing variables for COVID-19 patient’s complications (see Supplementary Table 4). In addition, COPD and cerebrovascular disease were pre-existing health conditions that contributed to elevated collinearity (see Supplementary Table 5). This suggested that complications in COVID-19 patients could be caused by pre-existing health conditions or changes in blood parameters.

### Heterogeneity and publication bias

**Figure 3:**
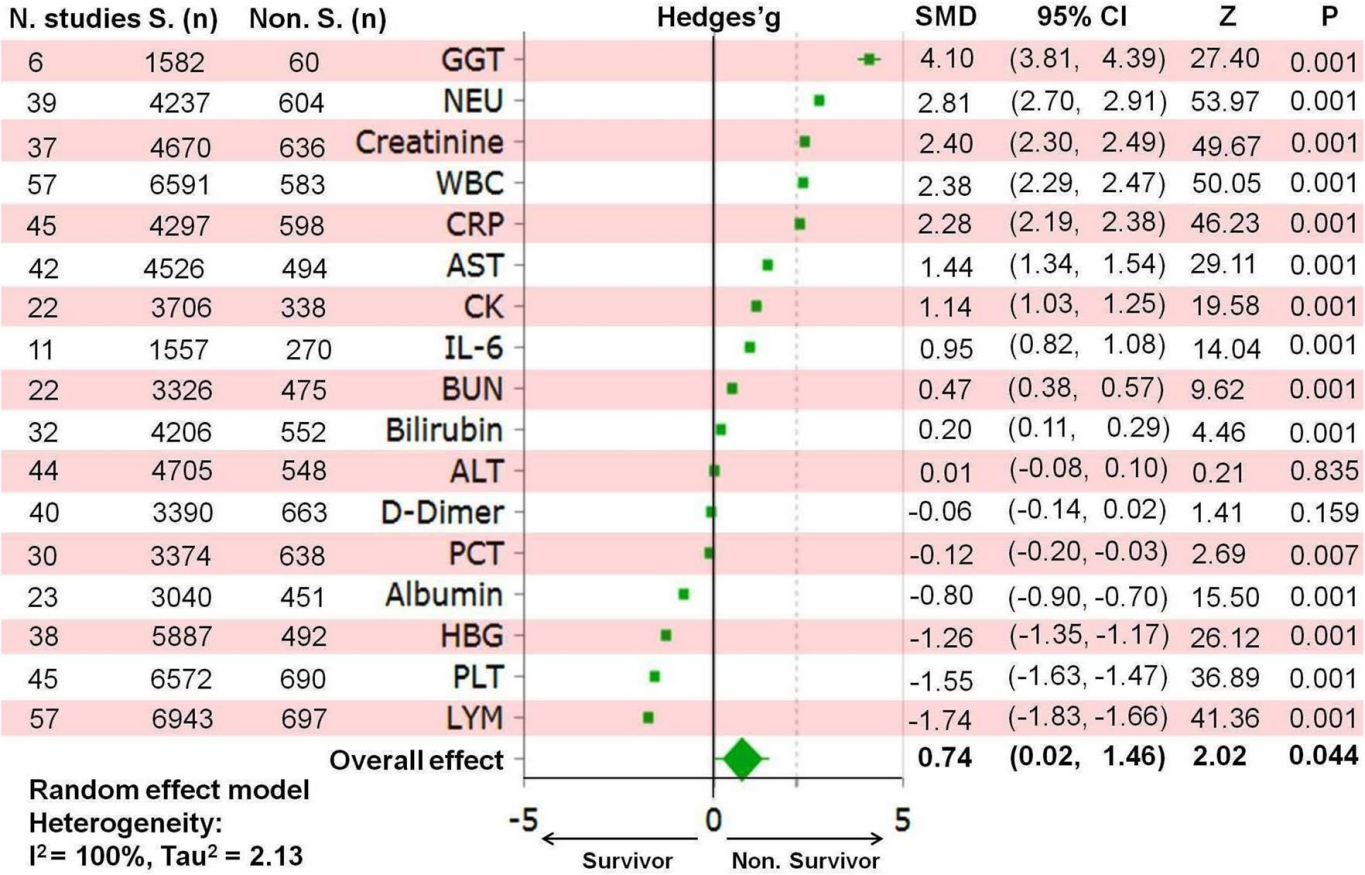
Forest plot of blood parameters in survivors and non-survivors of COVID-19. The Standardized Mean Difference (SMD) and the 95% confidence intervals (CIs) were used to define the effect size of different blood indices in survivors and non-survivors. S, survivors; GGT, gamma-glutamyl transferase; NEU, neutrophil; WBC, white blood cell; CRP, C-reactive protein; AST, aspartate aminotransferase; CK, creatine kinase; IL-6, interleukin-6; BUN, blood urea nitrogen; ALT, alanine aminotransferase; PCT, procalcitonin; HBG, hemoglobin; PLT, platelet; LYM, lymphocyte; n, population size.

**Figure 4:**
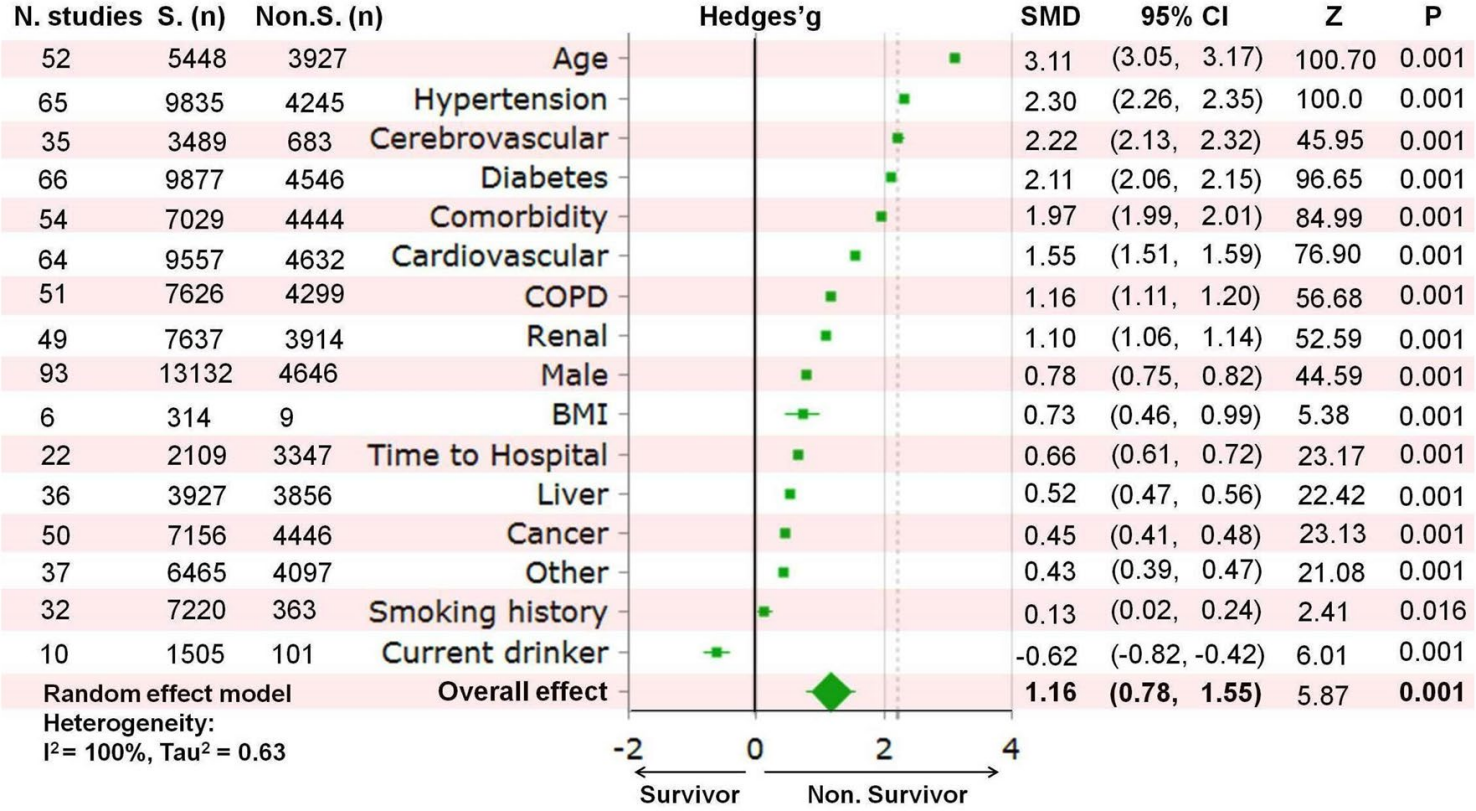
Forest plot of pre-existing health conditions in survivors and non-survivors of COVID-19. The Standardized Mean Difference (SMD) and the 95% confidence intervals (CIs) were used to define the prevalence of various risk factors and complications for survivors and non-survivors of COVID-19. Time to hospital, time from symptoms appearance to hospitalization; Cerebrovascular, cerebrovascular disease; Cardiovascular, cardiovascular disease; Renal, renal disease; Liver, liver disease; BMI, body mass index; COPD, chronic obstructive pulmonary disease; S, survivors; n, population size.

**Figure 5:**
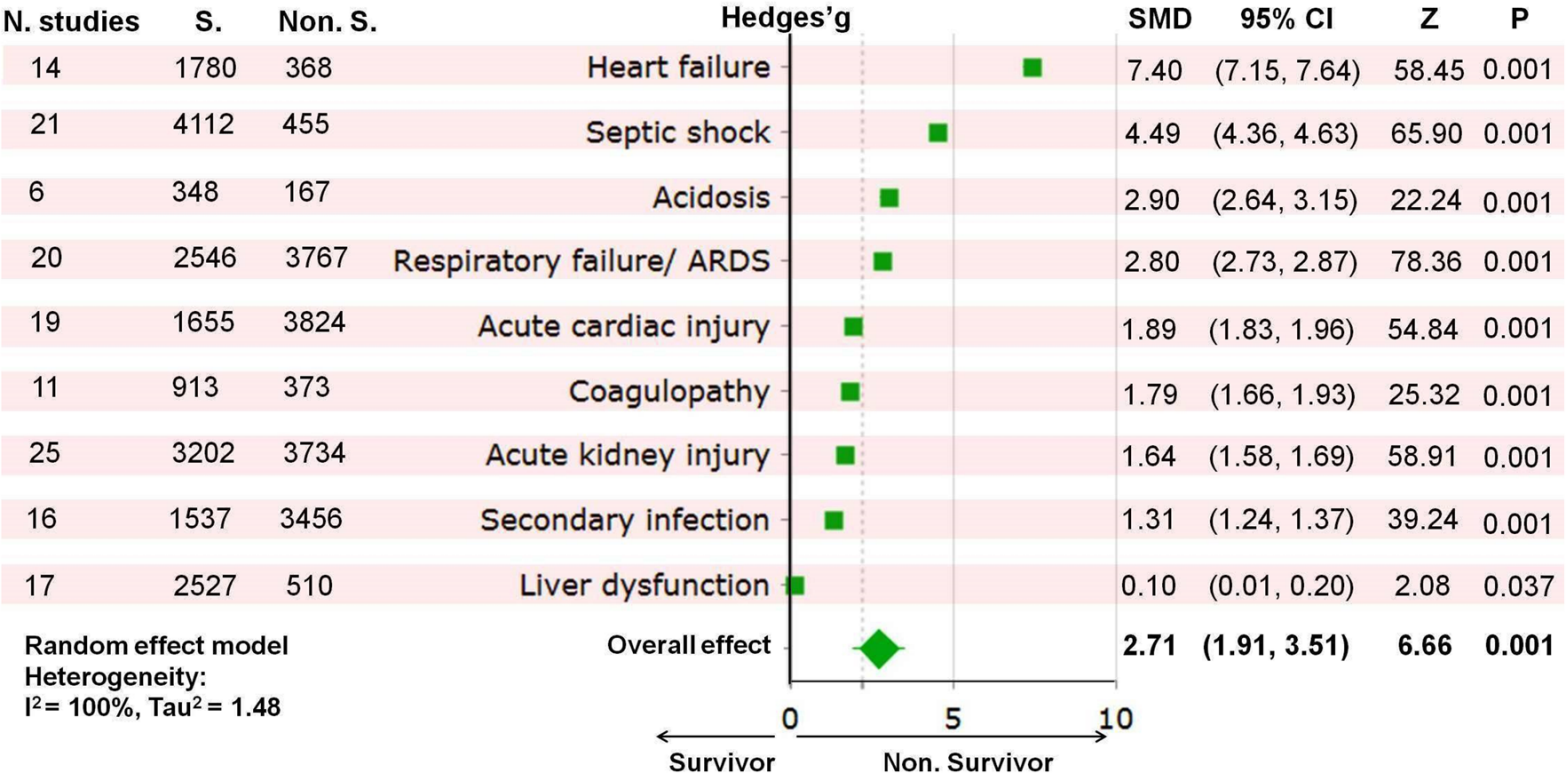
Forest plot of complications in survivors and non-survivors of COVID-19. The Standardized Mean Difference (SMD) and the 95% confidence intervals (CIs) were used to define the prevalence of various risk factors and complications for survivors and non-survivors of COVID-19. ARDS, acute respiratory distress syndrome; S, survivors; n, population size.

Clinical heterogeneity can lead to statistical heterogeneity, and can be observed using techniques like the I^2^ index or meta-regression^114^. The I^2^ index was high (100%) across all meta-analysis (Figs. 3, 4 and 5). In an attempt to better explain heterogeneity, multivariate meta-regression analysis revealed substantial heterogeneity in the outcome of interest (i.e. mortality and survival), which may be attributed to heterogeneity in exposure sources, such as blood indices, pre-existing conditions, or complications (Table 2).

**Table 2:** The Multivariate meta-regression analysis.

Variable	T	CI_95%_	*P*-value
Blood indices	1.85	(− 0.11, 1.60 )	0.083
Risk factors	4.77	(0.64, 1.68)	0.000
Complications	3.80	(1.07, 4.36 )	0.005

*CI* confidence interval.

Supplementary Table 6 shows the risk of bias assessment using QUIPS tool for all the observational studies used in the meta-analysis (n = 97). The majority of these 97 studies scored well (low risk of bias) in three of the QUIPS tool’s six domains: study attrition, prognostic factor measurement, and outcome measurement. The other three domains, namely study participation, study confounding and statistical analysis, were more problematic. According to the QUIPS tool, the majority of studies (74/97) used in this analysis had a moderate to high risk of bias.

### Quantitative synthesis of data

As shown in Table 2 and Appendix 2, the multivariate meta-regression analysis revealed that the risk factors (exposures) (t, 4.77; CI_95%_ [0.64, 1.68]; *P* < 0.001) were associated with the estimated intervention effect on COVID-19 mortality (outcome). Biochemical/hematological indices (t, 1.85; CI_95%_ [− 0.11, 1.60]; *P* = 0.083) tended to be associated with the estimated intervention effect on COVID-19 mortality. Moreover, complications were linked to the estimated intervention effect on COVID-19 mortality (t, 3.80; CI_95%_ [1.07, 4.36]; *P* = 0.005).

### Meta-analysis of individual hematological indices

Figure 3 displays the individual Hedges’g for each blood parameter, along with the corresponding CI_95%_. Since there was substantial statistical heterogeneity (*P* < 0.001), a random-effect model was used to evaluate the effect sizes (see Appendix 2).

Non-survivor COVID-19 had higher number of NEU (2.81[2.70, 2.91]; Z = 53.97; *P* < 0.001) and WBC (2.38 [2.29, 2.47]; Z = 50.05; *P* < 0.001) counts, as well as higher GGT (4.10 [3.81, 4.39]; Z = 27.40; *P* < 0.001), Cr (2.40 [2.30, 2.49]; Z = 49.67; *P* < 0.001), c-reactive protein (CRP) (2.28 [2.19, 2.38]; Z = 46.23; *P* < 0.001), aspartate aminotransferase (AST) (1.44 [1.34, 1.54]; Z = 29.11; *P* < 0.001), CK (1.14 (1.03, 1.25); Z = 19.58; *P* < 0.001), interleukin (IL) − 6 (0.95 [0.82, 1.08]; Z = 14.04; *P* < 0.001), BUN (0.47 [0.38, 0.57]; Z = 9.62; *P* < 0.001), and bilirubin (0.20 [0.11, 0.29]; Z = 4.46; *P* < 0.001) levels.

Compared to survivors, non-survivors had a smaller number of lymphocyte (LYM) (− 1.74 [− 1.83, − 1.66]; Z = 41.36; *P* < 0.001) and PLT (− 1.55 [− 1.63, − 1.47]; Z = 36.89; *P* < 0.001), as well as lower hemoglobin (− 1.26 [− 1.35, − 1.17]; Z = 26.12; *P* < 0.001), albumin (− 0.80 [− 0.90, − 0.70]; Z = 15.50; *P* < 0.001), and procalcitonin (− 0.12 [− 0.20, − 0.03]; Z = 2.69; *P* = 0.007) levels.

### COVID-19 mortality increases with age, hypertension, cerebrovascular disease, and diabetes

Figure 4 shows the meta-analysis forest plot in terms of pre-existing conditions based on the random effect model (see Appendix 2). Patient’s age (3.11 [3.05, 3.17]; Z = 100.70; *P* < 0.001); hypertension (2.30 (2.26, 2.35); Z = 100.00; *P* < 0.001), cerebrovascular disease (2.22 [2.13, 2.32]; Z = 45.95; *P* < 0.001), diabetes (2.11 [2.06, 2.15]; Z = 96.66; *P* < 0.001), any comorbidities (1.97 [1.99, 2.01]; Z = 84.99; *P* < 0.001), cardiovascular disease (1.55 [1.51, 1.59]; Z = 76.90; *P* < 0.001), COPD (1.16 [1.11, 1.20]; Z = 56.68; *P* < 0.001), renal disease (1.10 [1.06, 1.14]; Z = 52.59; *P* < 0.001), male sex (0.78 [0.75, 0.82]; Z = 44.59; *P* < 0.001), body mass index (BMI) (0.73 [0.46, 0.99]; Z = 5.38; *P* < 0.001), time from symptoms appearance to hospitalization (0.66 [0.61, 0.72]; Z = 23.17; *P* < 0.001), liver disease (0.52 [0.47, 0.56]; Z = 22.42; *P* < 0.001), cancer (0.45 [0.41, 0.48]; Z = 23.13; *P* < 0.001) and smoking history (0.13 [0.02, 0.24]; Z = 2.41; *P* = 0.016) was higher among non-survivors.

### Meta-analysis identifies common complications among COVID-19 non-survivors

The prevalence of current complications was higher in COVID-19 non-survivors (2.71 [1.91, 3.51]; Z = 6.66; *P* < 0.001; I^2^ = 100.0%; Tau^2^ = 1.48) than survivors (see Fig. 5 and Appendix 2). Heart failure was the most common complication in COVID-19 non-survivors (7.40 [7.15, 7.64]; Z = 58.45; *P* < 0.001), followed by septic shock (4.49 [4.36, 4.63]; Z = 65.90; *P* < 0.001), acidosis (2.90 [2.64, 3.15]; Z = 22.24; *P* < 0.001), respiratory failure (2.80 [2.73, 2.87]; Z = 78.36; *P* < 0.001), acute cardiac injury (1.89 [1.83, 1.96]; Z = 54.84; *P* < 0.001), coagulopathy (1.79 [1.66, 1.93]; Z = 25.32; *P* < 0.001), acute kidney injury (1.64 [1.58, 1.69]; Z = 58.91; *P* < 0.001), secondary infection (1.31 [1.24, 1.37]; Z = 39.24; *P* < 0.001), and liver dysfunction (0.10 [0.01, 0.20]; Z = 2.08; *P* = 0.037).

### Network analysis supports the results of the meta-analysis

At a cutoff point of 50%, the network correlation for blood indices (Fig. 2a), risk factors (Fig. 2b), and complication (Fig. 2c) was demonstrated. The number of PLT and LYM, as well as hemoglobin concentration, was associated with COVID-19 survivors at a maximum cutoff point of 72% where all parameters were disconnected after this cutoff point (Fig. 2d). At a maximum cutoff point of 93%, the number of NEU, GGT concentration, and the incidence of COVID-19 mortality were all related (Fig. 2e).

COVID-19 mortality was discovered to be linked to patient’s age, hypertension, cerebrovascular disease, diabetes, any comorbidities, cardiovascular disease (as pre-existing conditions, at a maximum cutoff point of 79%, Fig. 2f) and heart failure (as a complication, at a maximum cutoff point of 97%, Fig. 2g), according to the network analysis. These findings corroborated the results of the meta-analysis. The network analysis was able to map the association between all parameters, including mortality, blood indices, complications and pre-existing conditions, while the meta-analysis only ranked the potent factors involved in COVID-19 mortality.

### Clinical outcomes and meta-analysis of COVID-19-infected ICU/non-ICU patients

The clinical outcomes of ICU-admitted COVID-19 patients and non-ICU COVID-19 patients are shown in Table 3. The average age of ICU survivors (n = 336) and non-survivors (n = 201) was 59.5 years (CI_95%_: 50.1, 68.9) and 70.7 years (CI_95%_: 65.5, 76.0), respectively. Non-ICU survivors (n = 2112) had an average age of 43.6 years (CI_95%_: 38.5, 48.7). Males outnumbered females in the ICU non-survivors (63.7%), ICU survivors (50.9%), and non-ICU survivors (52.3%). Diarrhea, nausea, coughing, and dyspnoea were more common in ICU-admitted patients than in non-ICU patients. In comparison to ICU non-survivors, a greater proportion of ICU survivors received antibiotics (83.5 vs. 62.5%), corticosteroids (83.0 vs. 54.5%), and intravenous immunoglobin (51.4 vs. 20.5%).

**Table 3:** Characteristics, blood parameters, symptoms, and pre-existing conditions among ICU and non-ICU patients infected with COVID-19.

Characteristic	ICU only (n = 537)		Non-ICU only(n = 2112)
	Survivors (n = 336, 62.6%)	Non-survivors (n = 201, 37.4%)	Survivors (n = 2112)
Age—no, yr, mean (CI_95%_)	336, 59.5 (50.1, 68.9)	201, 70.7 (65.5, 76.0)	2112, 43.6 (38.5, 48.7)
Male—no./total no. (%)	171/336 (50.9)	128/201 (63.7)	1120/2112 (53.0)
Drinker (Yes)—no./total no. (%)	3/30 (10.0)	NR	NR
Smoker (Yes)—no./total no. (%)	2/30 (5.0)	23/176 (13.1)	52/653 (8.0)
Hypertension (Yes)—no./total no. (%)	179/326 (54.9)	88/176 (50.0)	196/613 (32.0)
Diabetes (Yes)—no./total no. (%)	154/326 (47.2)	45/176 (25.6)	123/752 (16.4)
Cardiovascular disease (Yes)—no./total no. (%)	89/323 (27.5)	29/176 (16.5)	88/719 (12.2)
Chronic obstructive lung (COPD) (Yes)—no./total no. (%)	17/237 (7.2)	19/176 (10.8)	76/795 (9.6)
Cancer (Yes)—no./total no. (%)	17/86 (19.8)	144/176 (81.8)	35/1763 (2.0)
Liver disease (Yes)—no./total no. (%)	17/86 (19.8)	9/144 (6.3)	46/328 (14.0)
Cerebrovascular disease (Yes)—no./total no. (%)	NR	16/176 (9.1)	21/1399 (1.5)
Renal disease (Yes)—no./total no. (%)	0/86 (0.0)	6/176 (3.4)	48/688 (7.0)
Other diseases (Yes)—no./total no. (%)	NR	13/32 (40.6)	122/448 (27.2)
Treatment			
Antibiotic—no./total no. (%)	91/109 (83.5)	20/32 (62.5)	748/1707 (43.8)
Antiviral (Any)—no./total no. (%)	102/119 (85.7)	119/144 (82.6)	1390/1814 (76.6)
Corticosteroid*—no./total no. (%)	88/106 (83.0)	96/176 (54.5)	307/1706 (18.0)
Intravenous immunoglobin—no./total no. (%)	56/109 (51.4)	36/176 (20.5)	357/1642 (21.7)
Noninvasive mechanical ventilation—no./total no. (%)	52/89 (58.4)	54/176 (30.7)	206/1917 (10.7)
Invasive mechanical ventilation—no./total no. (%)	248/306 (81.0)	35/176 (19.9)	142/1832 (7.8)
Ethnics			
White, European—no./total no. (%)	25/336 (7.4)	25/201 (12.4)	0/0 (0.0)
African American—no./total no. (%)	159/336 (47.3)	0/0 (0.0)	0/0 (0.0)
Asian—no./total no. (%)	128/336 (38.0)	174/201 (87.6)	2082/2082 (100.0)
Hispanic, Latino—no./total no. (%)	0/0 (0.0)	0/0 (0.0)	0.0 (0.0)
Multi-ethnicity—no./total no. (%)	24/336 (7.1)	0/0 (0.0)	0/0 (0.0)
Hematological indices—n, mean 10^9^/L (CI_95%_)			
White blood cells	119, 4.95 (4.55, 5.35)	176, 6.15 (3.37, 8.94)	1899, 6.26 (5.15, 7.37)
Lymphocyte	119, 0.96 (0.28, 1.65)	176, 0.66 (0.10, 1.21)	2038, 1.04 (0.79, 1.29)
Platelet	119, 164.94 (71.54, 258.34)	176, 154.17 (36.42, 271.91)	1974, 176.2 (166.0, 186.37)
Neutrophil	99, 3.63 (2.83, 4.43)	176, 4.96 (0.72, 9.19)	1367, 4.97 (3.32, 6.61)
Biochemical indices—n, mean (CI_95%_)			
Hemoglobin—g/L	33, 138.01 (133.52, 142.49)	144, 115.82 (83.04, 148.60)	1050, 124.17 (107.85, 140.5)
Albumin—g/L	109, 35.81 (20.50, 51.12)	144, 34.53 (21.25, 47.80)	1318, 38.82 (30.61, 47.02)
Alanine aminotransferase—U/L	119, 21.41 (12.20, 30.62)	144, 28.50 (22.15, 34.85)	1691, 23.99 (20.21, 27.76)
Aspartate aminotransferase—U/L	119, 24.74 (9.84, 43.63)	144, 32.25 (− 53.3, 118.01)	1460, 32.16 (24.94, 39.38)
Total bilirubin—µmol/L	116, 13.98 (− 3.94, 31.90)	176, 10.48 (8.19, 12.78)	1305, 12.24 (8.57, 15.92)
Blood urea nitrogen—mmol/L	99, 4.33 (1.68, 7.01)	176, 6.78 (− 20.1, 33.59)	1406, 6.40 (4.35, 8.44)
Creatinine—µmol/L	119, 64.97 (60.53, 69.41)	176, 72.90 (52.40, 93.41)	1588, 74.60 (68.15, 81.05)
Creatine Kinase—U/L	119, 62.63 (52.41, 72.84)	NR	1572, 80.44 (37.77, 123.10)
C-reactive protein—mg/L	336, 63.77 (− 49.36, 176.90)	176, 75.47 (− 84.23, 235.17	1410, 31.72 (1.32, 62.12)
Procalcitonin—ng/mL	119, 0.05 (− 0.003, 0.10)	174, 0.40 (− 3.67, 4.47)	654, 0.17 (0.08, 0.26)
D-dimer—mg/L	33, 0.34 (0.11, 0.57)	32, 0.62 (NA)	1680, 2.18 (0.75, 3.62)
Symptoms			
Fever—no./total no. (%)	110/119 (92.4)	132/176 (75.0)	1622/2038 (79.6)
Cough—no./total no. (%)	94/119 (79.0)	126/176 (71.6)	1249/2038 (61.3)
Shortness of Breath (dyspnoea)—no./total no. (%)	56/119 (47.1)	110/176 (62.5)	219/1618 (13.5)
Sputum—no./total no. (%)	45/119 (37.8)	61/174 (35.1)	503/1632 (30.8)
Myalgia—no./total no. (%)	7/33 (21.2)	11/174 (6.3)	138/1725 (8.0)
Fatigue—no./total no. (%)	10/33 (30.3)	84/176 (47.7)	309/719 (43.0)
Diarrhea—no./total no. (%)	18/119 (15.1)	21/174 (12.1)	135/1727 (7.8)
Nausea (Vomiting)—no./total no. (%)	1/13 (7.7)	29/174 (16.7)	121/1913 (6.3)

*CI* confidence interval, *NR* not reported, *NA* not applicable.

**Figure 6:**
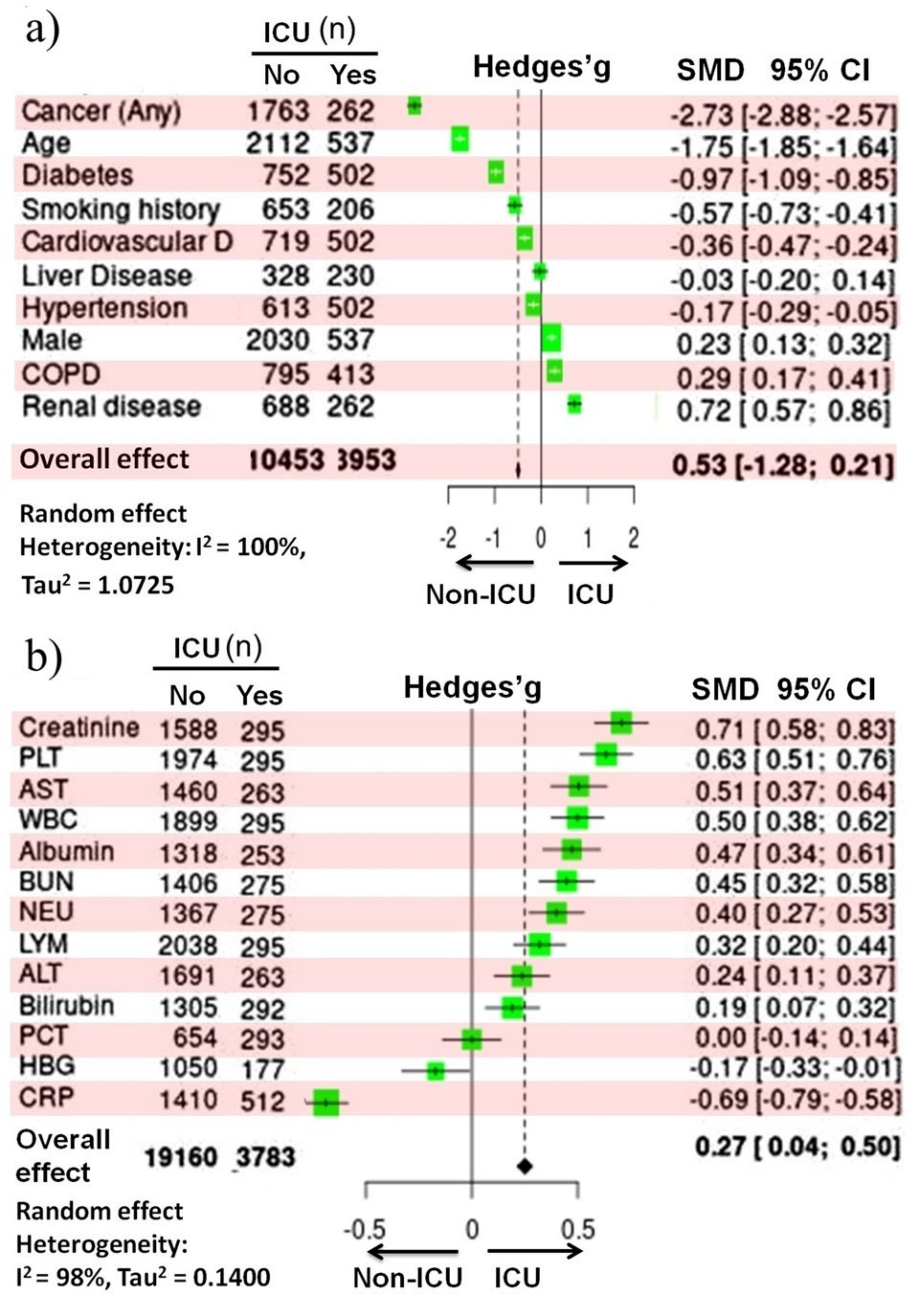
Forest plot of (**a**) pre-existing health issues and (**b**) blood parameters in COVID-19-infected ICU and non-ICU patients. The Standardized Mean Difference (SMD) and the 95% confidence intervals (CIs) were used to define the effect size. n, population size; D, diseases; COPD, chronic obstructive pulmonary disease; S, survivors; NS, non-survivors; ALT, alanine aminotransferase; WBC, white blood cell; PCT, procalcitonin; BUN, blood urea nitrogen; NEU, neutrophil; AST, aspartate aminotransferase; CRP, C-reactive protein; PLT, platelet; LYM, lymphocyte; HBG, hemoglobin.

Figure 6 shows the individual Hedges'g in terms of pre-existing conditions and blood parameters in ICU and non-ICU patients, as well as the corresponding CI_95%_. Because there was significant statistical heterogeneity (*P* < 0.001), the effect sizes were evaluated using a random-effect model. The prevalence of renal disease (0.72 [0.57, 0.86]; Z = 9.64; *P* < 0.001), COPD (0.29 [0.17, 0.41]; Z = 4.74; *P* < 0.001), and male percentage (0.23 [0.13, 0.32]; Z = 4.63; *P* < 0.001) were greater in COVID-19 patients admitted to ICU (Fig. 6a). Cancer was the most common in non-ICU patients (− 2.73 [− 2.88, − 2.57]; Z = − 34.55; *P* < 0.001), followed by age (− 1.75 [− 1.85, − 1.64]; Z = − 32.34; *P* < 0.001), and diabetes (− 0.97 [− 1.09, − 0.85]; Z = − 15.97; *P* < 0.001, Fig. 6a).

Platelet (0.63 [0.51, 0.76]; Z = 10.06; *P* < 0.001), WBC (0.50 [0.38, 0.62]; Z = 7.92 *P* < 0.001), and NEU counts (0.40 [0.27, 0.53]; Z = 6.03; *P* < 0.001) were observed to be linked with ICU referral (Fig. 6b). Creatinine levels (0.71 [0.58, 0.83]; Z = 10.97; *P* < 0.001), AST activity (0.51 [0.37, 0.64]; Z = 7.49; *P* < 0.001), albumin levels (0.47 [0.34, 0.61]; Z = 6.82; *P* < 0.001), and BUN levels (0.45 [0.32, 0.58]; Z = 6.75; *P* < 0.001) were also observed to be linked with ICU referral (Fig. 6b). It was found that CRP levels (− 0.69 [− 0.79, − 0.58]; Z = − 13.04; *P* < 0.001) and HBG levels (− 0.17 [− 0.33, − 0.01]; Z = − 2.10; *P* < 0.001) preferred non-ICU patients (Fig. 6b).

**Figure 7:**
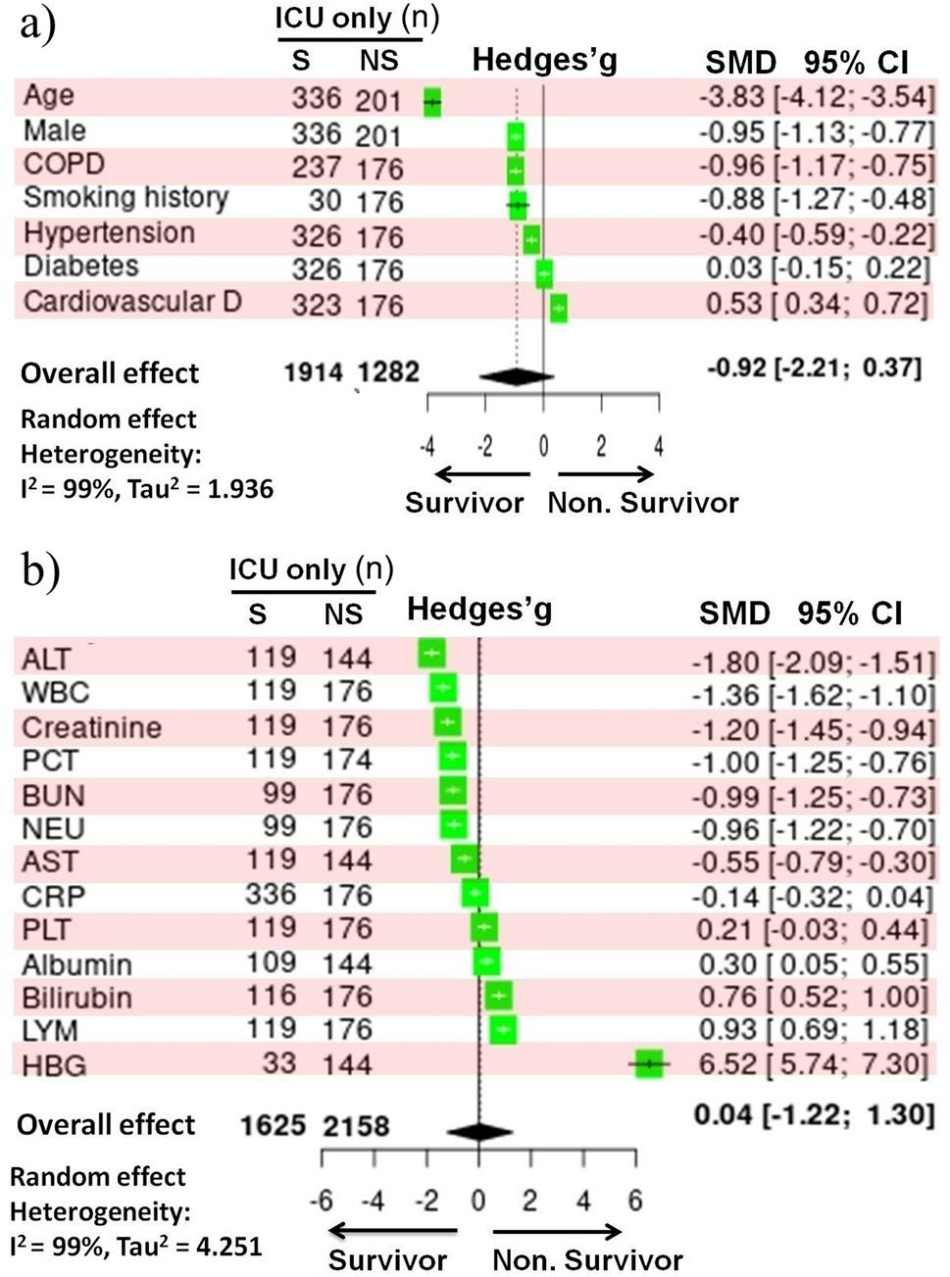
Forest plot of (**a**) pre-existing health conditions and (**b**) blood parameters in survivors and non-survivors of COVID-19 patients admitted to the intensive care unit (ICU). The Standardized Mean Difference (SMD) and the 95% confidence intervals (CIs) were used to define the effect size. n, population size; D, diseases; COPD, chronic obstructive pulmonary disease; S, survivors; NS, non-survivors; ALT, alanine aminotransferase; WBC, white blood cell; PCT, procalcitonin; BUN, blood urea nitrogen; NEU, neutrophil; AST, aspartate aminotransferase; CRP, C-reactive protein; PLT, platelet; LYM, lymphocyte; HBG, hemoglobin.

Age (− 3.83 [− 4.12, − 3.54]; Z = − 25.99; *P* < 0.001) favored survival among ICU-admitted patients, while cardiovascular disease (0.53 [0.34, 0.72]; Z = 5.55; *P* < 0.001) was frequent among ICU non-survivors (Fig. 7a). Compared to ICU survivors, ICU non-survivors had a greater number of WBC (− 1.36 [− 1.62, − 1.10]; Z = − 10.35; *P* < 0.001) and NEU (− 0.96 [− 1.22, − 0.70]; Z = − 7.26; *P* < 0.001). Also, ICU non-survivors had higher alanine aminotransferase (ALT, − 1.80 [− 2.09, − 1.51]; Z = − 12.25; *P* < 0.001), Cr (− 1.20 [− 1.45, − 0.94]; Z = − 9.29; *P* < 0.001), PCT (− 1.00 [− 1.25, − 0.76]; Z = − 7.97; *P* < 0.001), BUN (− 0.99 [− 1.25, − 0.73]; Z = − 7.46; *P* < 0.001), and AST (− 0.55 [− 0.79, − 0.30]; Z = − 4.33; *P* < 0.001) (Fig. 7b). However, lower levels of HBG (6.52 [5.74, 7.30]; Z = 16.30; *P* < 0.001), total bilirubin (0.76 [0.52, 1.00]; Z = 6.14; *P* < 0.001) and albumin (0.30 [0.05, 0.55]; Z = 2.38; *P* = 0.02), as well as LYM (0.93 [0.69, 1.18]; Z = 7.46; *P* < 0.001), favored mortality in ICU-admitted (ICU non-survivors; Fig. 7b).

## Discussion

According to the findings of this study, certain hematological/biochemical markers, pre-existing conditions, and complications were associated with mortality in COVID-19 patients, particularly in ICU-admitted patients, and should be taken into account for patient care. Furthermore, a multilevel approach based on network analysis and correlation analysis will aid in the deeper understanding of interactions within and between exposures and outcomes.

Our meta-analysis revealed that COVID-19 non-survivors, as well as ICU non-survivors, had higher NEU and greater LYM counts. According to the study of Qin et al^115^, lymphopenia (low LYM counts) and an increased NEU–LYM ratio were frequently observed in patients with severe COVID-19. This was also a more common characteristic in COVID-19-related death^12^. Inflammatory mediators, such as IL-2 and IL-6, can cause serious lymphopenia, resulting in LYM loss^115^. Qin et al.^115^ indicated that SARS-CoV-2 infection affects LYMs, resulting in secondary bacterial infections and an increased NEU count. Indeed, neutrophilia (NEU count > 7.5 × 10^9^/L) has been linked to bacterial inflammation, cytokine storm, and hyper-inflammation^116^, all of which play significant pathogenetic roles in COVID-19 infection^115,117^. Consistent with previous studies^32^, we identified an increase in WBC and NEU counts and a decrease in LYM in COVID-19 non-survivors, particularly in ICU non-survivors. Therefore, changes in WBC, NEU, and LYM counts were associated with the risk of mortality in COVID-19 patients and patients admitted to the ICU.

In this meta-analysis, we found that GGT and AST concentrations were higher in COVID-19 non-survivors. Furthermore, ALT and AST levels were greater in ICU patients, as well as ICU non-survivors. Concentrations of ALT, AST, and GGT have been found to be markedly greater in dead patients than in recovered patients^32,118^. In a previous study^119^, GGT levels were shown to be elevated in COVID-19 patients. Higher GGT levels were associated with lower albumin and higher CRP, ALT, and ALP levels in the 82 COVID-19 patients who did not have chronic liver disease or an alcohol history. This elevation suggests that the liver is involved in COVID-19 patients^120^. Bernal-Monterde et al.^119^ reported that increased levels of GGT and ALP, as well as decreased albumin levels, were associated with increased risk of death in COVID-19. Indeed, viral infections that commonly affect the respiratory tract cause hypoxia^119^. In patients with pandemic H1N1 influenza infection, serum levels of ALT, AST, and GGT were found to be positively correlated with hypoxia^121^. These findings were consistent with our Pearson analysis, which found a strong relationship between GGT levels and respiratory failure (r = 0.91) or heart failure (r = 0.88), but not with liver dysfunction (r = 0.14). As mentioned above, heart failure was found to be the most common complications in non-survivors. Interestingly, Spearman correlation showed significant and non-linear associations between GGT and ALT and liver dysfunction. Increased ALT, AST, and GGT levels in COVID-19 patients, particularly in ICU non-survivors, appeared to be caused by heart failure-induced hypoxia, although further research is required to understand the details.

Since meta-analysis cannot establish relationships between variables, bivariate analysis, such as Spearman and Pearson correlation, and VIF were used to determine relationships and multicollinearity among influencing factors (i.e., blood indices and pre-existing conditions). Data showed that some complications found in COVID-19 patients, such as heart failure, were correlated with pre-existing conditions (i.e., age), as well as lower PLT and LYM counts or higher GGT levels. Moreover, network analysis was used to visualize the structure of relationships between factors affecting the COVID-19 outcome. Indeed, this approach will help in explaining the relationships between variables like blood indices, pre-existing conditions and complications, as well as the relationships between these variables and the outcome, i.e., mortality and survival. Network analysis identified a link between heart failure and increased mortality in COVID-19 patients. Therefore, combining a multi-level analysis with meta-analysis would help to achieve a better understanding of the relationships between patient characteristics and outcome.

This meta-analysis revealed a greater concentration of GGT, BUN, and Cr in COVID-19 non-survivors. Furthermore, COVID-19 patients admitted to the ICU had higher Cr levels and a greater prevalence of renal disease. Creatinine and BUN levels were also higher in died ICU patients. These findings suggest that SARS-CoV-2 has a clear impact on human kidneys^122^ and increases COVID-19 patient referral to the ICU, as well as ICU mortality. A study of 701 patients revealed that elevated serum Cr levels on admission were associated with severity due to severe coagulation pathway abnormalities^123^. Furthermore, increased urea levels had comparable, if not greater, impacts on hazard ratios. Another kidney failure marker is GGT^124^, which is a cell-surface enzyme that metabolizes extracellular glutathione, the primary antioxidant in mammalian cells^125^. A high level of GGT is often regarded as an early and marker of oxidative stress^126^ and it can be a source of reactive oxygen species in the presence of iron^126,127^. This, in turn, may result in renal vasoconstriction, salt retention, and subsequent kidney damage^128^. Abnormalities in the routine urine test performed on admission have been linked to disease progression and an increased risk of in-hospital death^129^. As a result, renal abnormalities on admission revealed a greater risk of deterioration, requiring proper triaging^129^; further research is needed.

Current evidence indicates that complications in COVID-19 patients may be caused by the virus’s direct effect, immune-mediated inflammation or drug-induced toxicity, assuming that the majority of patients were given high doses of antibiotics, antiviral drugs, and steroids^118^. Bivariate analyses showed that some complications in COVID-19 patients (e.g., heart failure, septic shock, respiratory failure, acute cardiac and kidney injury, and secondary infections) were mainly associated in a non-linear manner with both pre-existing conditions (such as age, comorbidities, diabetes, and cerebrovascular disease) and blood parameters (such as PLT, CK, and IL-6). However, it is unclear to what extent complications are exacerbated by COVID-19 infection. Zhou et al.^12^ found that sepsis was the most common complication, followed by respiratory failure, ARDS, and heart failure. In our meta-analysis, heart failure and septic shock were the most common complications diagnosed in dead patients. Sahu et al.^130^ found that COVID-19 patients who died from infection had a gradual increase in CRP levels. Li et al.^131^ suggested that direct viral disruption, hyper-inflammation, and hypoxemia may all contribute to cardiac injury. Serum CRP, as an inflammatory marker, has been linked to disease severity^132^, lung lesions^133^, acute kidney damage^134^, and cardiac injuries^135^ in COVID-19 patients. Furthermore, we found that CRP levels were greater in COVID-19 ICU patients than in non-ICU patients. Our findings imply that CRP may be a biomarker of ICU referral, as well as death in COVID-19 patients, emphasizing the need of regularly monitoring CRP changes.

According to the present meta-analysis, COVID-19 patients admitted to the ICU and COVID-19 non-survivors had a lower PLT counts, as well as lower hemoglobin and albumin concentrations. Our findings corroborated the previous study that showed a decrease in the number of PLTs in non-survivors but an increase in survivors^136^. Zhao et al.^136^ found that PLT count may dramatically reflect pathophysiological changes in COVID-19 patients, and an early decrease in PLT was associated with COVID-19 mortality. Viral infection appears to have damaged lung tissue, resulting in PLT activation, aggregation, and entrapment, which lead to thrombosis and increased PLT consumption^136^. PLTs have a short life cycle (8–10 days) and very few PLTs are preserved in bone marrow;^137^ it may be responsive to the severity of the patient’s conditions. Furthermore, viruses may cause a decrease in PLT production as a result of megakaryocyte infection, which may contribute to megakaryocyte apoptosis^138^. Therefore, PLT measurement may be beneficial in the care of COVID-19 patients and ICU referral, resulting in a much earlier and more effective prognosis.

Liu et al.^139^ reported that COVID-19 patients had the most consistent decreases in hemoglobin levels. The first case of COVID-19 in the United States revealed a minor decrease in hemoglobin on day 6 of illness^30^. Notably, patients with a composite outcome (i.e., ICU admission, invasive ventilation, and death) had lower hemoglobin levels^18^. The present meta-analysis identified a link between low hemoglobin levels in COVID-19 patients admitted to the ICU and ICU mortality (Figs. 6b and 7b). It is worth noting that the atypical type of ARDS in COVID-19 patients causes inadequate blood oxygenation and can be fatal^140^. Because hemoglobin concentration in the blood is one of the most significant factors of the oxygen-carrying capacity of the blood, hemoglobin levels in COVID-19 patients may play a key role in this respect^140^. Anemia in ICU patients has been linked to an increased risk of death. It has also been linked to a higher risk of complications such as acute kidney injury^141^. Non-survivors' hemoglobin levels upon admission and throughout the last ICU days are much lower than survivors'^140^. Similar to our findings, it was recently reported that patients with COVID-19 have considerably lower hemoglobin levels than patients that were not admitted to ICU^142^. Dinevari et al.^143^ reported that, the frequency of mortality (anemic: 23.9% vs. non-anemic: 13.8%) and ICU admission (anemic: 27.8% vs. non-anemic: 14.71%) were significantly greater in anemic COVID-19 patients than in non-anemic COVID-19 patients. It has been shown that inflammation caused by SARS-CoV-2 can disrupt erythropoiesis and decrease hemoglobin production. For example, IL-6 has been shown to be elevated in severe COVID-19 infection^117^ and disrupts hemoglobin production^144^. The present meta-analysis revealed that COVID-19 non-survivors had higher levels of IL-6. Our findings suggested that lower hemoglobin levels may be attributed to higher levels of IL-6, which requires further study in COVID-19 patients.

Age, hypertension, cerebrovascular disease, and diabetes have been found to be common risk factors among non-survivors in our meta-analysis. In accordance with the previous study^12^, we noticed that non-survivors were older than survivors (46.6 vs. 76.5 years), ICU survivors were older than non-ICU survivors (43.6 vs. 59.5 years), and ICU non-survivors were older than ICU survivors (70.7 vs. 59.5 years). Additionally, Spearman analysis showed that age had a linear relationship with heart failure but a nonlinear relationship with complications like respiratory failure, septic shock, and acute cardiac injury in COVID-19 patients. Furthermore, COVID-19 non-survivors had a greater proportion of hypertension and diabetes than survivors. ACE2 has been shown to be over-expressed in diabetic or hypertensive patient^145,146^. Diabetes and hypertension are treated with ACE inhibitors and angiotensin II type-I receptor blockers (ARBs), which causes an increase in ACE2 expression and infection with COVID-19^146,147^. According to previous research, the re-admission rate to the ICU for sepsis was twice as high in the ACE inhibitor (1.08%) and ARB (1.04%) groups than in the non-user group^148^. The findings of our Spearman analysis showed a significant correlation between diabetes and hypertension with septic shock as a complication of COVID-19 infection. Moreover, another study found a link between cerebrovascular disease and the risk of death in COVID-19 patients^149^, which was consistent with our findings. SARS-CoV-2 has been shown to have neuro-invasive properties and the ability to spread from the respiratory system to the central nervous system^150^. COVID-19 can also cause cerebrovascular complications as a result of inflammation, hypoxia, and diffuse intravascular coagulation^151^. Therefore, pre-existing conditions, such as age, cerebrovascular disease, diabetes and hypertension, may lead to a greater risk of death in COVID-19 patients. Furthermore, we found that cardiovascular disease, renal disease, age, and gender were connected to increased ICU referral and mortality. Thus, COVID-19 patients with these pre-existing conditions should be closely monitored.

We found that exposures, i.e., demographic factors (e.g., age, gender, smoking, and alcohol consumption), as well as pre-existing conditions or comorbidities, were the primary sources of heterogeneity in this study. This may be due to inconsistencies in study designs, large differences in sample size, and differences in study characteristics. In this study, we focused on a large particular subgroup (e.g., survivors or non-survivors, which included patients of various ages, genders, and pre-existing health conditions). This basically results in heterogeneity as confirmed by I^2^ index and multivariate meta-regression analysis. Moreover, the high heterogeneity in this meta-analysis may be explained by studies that reported either individual patient data or the mean for a cohort of patients. Other factors such as heterogeneity in survival group (which included mild to severe cases) and non-survival groups (who had various treatments) can also lead to publication bias^152^.

It should also be noted that the sample size of the studies used for this meta-analysis ranged from 1 to 3200 cases (mean: 132 [CI_95%_: 75.0, 189.0]). In terms of variability in blood sample collection, it was observed that most blood samples were collected around the time of admission The interval between the onset of symptoms and hospitalization ranged from 0 to 18 days, with an average of 7.0 (CI_95%_: 6.4, 7.5) days. As a result, obtaining blood samples and recording symptoms at various stages of the infection at the time of admission may obviously affect outcomes; this difference in data collection period may contribute to significant heterogeneity in samples. Furthermore, the period of follow-up in the studies used in this meta-analysis differed from 6 days to 4 weeks; such changes in follow-up times may impact disease development and, therefore, corresponding outcomes^13^.

According to the QUIPS assessment, the majority of the studies used had a moderate risk of bias. The majority of the studies included in this meta-analysis lacked data on the impact of blood parameters and pre-existing conditions on comorbidities, as well as the relationship between such comorbidities and mortality. QUIPS assessment suggested that future research should consider experiments with adequate statistical power and appropriate statistical methods to address the potential interrelationships between all prognostic factors, complications, and outcomes in COVID-19 patients.

In this study, we used a multi-level approach, including meta-analysis, bivariate analysis and network analysis, to establish potential associations between exposures (e.g., patient characteristics) and outcomes (e.g., mortality or survival). However, this meta-analysis has several limitations. We did not perform sensitivity and subgroup analyses, despite the inclusion of studies with patients at various stages of COVID-19. Moreover, the data were obtained from a variety of countries, including developed and developing nations, with varying levels of medical facilities, suggesting different management guidelines for related medical comorbidities.

In conclusion, various pre-existing conditions and biochemical/hematological indices were related with an increased risk of ICU referral and mortality in COVID-19 patients, particularly those admitted to the ICU. Also, the data showed that complications, such as heart failure and septic shock, were more common in COVID-19 non-survivors, which could be attributed to patient characteristics, emphasizing the importance of pre-screening at triage^153^.

## Supplementary Information

Supplementary Information 1.

Supplementary Information 2.

Supplementary Information 3.

## Data Availability

The datasets used and/or analyzed during the current study available from the corresponding author on reasonable request.
