# Supplementary material for: Risk of mortality in COVID-19 patients: a meta- and network analysis
Source: Sci Rep. 2023 Feb 6;13:2138. doi: 10.1038/s41598-023-29364-8 (PMC9901837; doi:10.1038/s41598-023-29364-8)
Supplement: Supplementary file 1 — Supplementary Information 1. [file 41598_2023_29364_MOESM1_ESM.pdf]

# Risk of mortality in COVID-19 patients: a meta- and network analysis

Rasoul Kowsar[1*], Amir Mohammad Rahimi[2], Magdalena Sroka[3], Alireza Mansouri[4] , Khaled Sadeghi[1], Elham Bonakdar[1], Sayed Farshad Kateb[5], Amir Hossein Mahdavi[1]

[1] Department of Animal Sciences, College of Agriculture, Isfahan University of Technology, Isfahan, 84156–83111, Iran.

[2] Department of Developmental Biology, Göttingen Center for Molecular Biosciences (GZMB), Georg-August-University, Göttingen, 37073, Germany.

[3] University Medical Center Göttingen, Georg-August-University, Göttingen, 37075, Germany.

[4] Global Agromedicine Research Center (GAMRC), Obihiro University of Agriculture and Veterinary Medicine, Obihiro, Hokkaido, Japan.

[5] Yektadam Persian Co. Isfahan, Iran.

***Corresponding author**: Rasoul Kowsar, Rasoul_kowsarzar@yahoo.com. Isfahan University of Technology, Iran. Tel: +989177525210, PC: 8415683111.

**Supplementary Table 1.** Characteristics of included studies in the meta-analysis.

| Author | Country | Patients (N) | Male | Severity | Study type | Study length (days) | Survivors | Any Treatment |
|---|---|---|---|---|---|---|---|---|
| Yang et al. [4] | China | 52 | 67.0 | Severe | Observational | 33 | Both | No |
| Zhou et al. [12] | China | 191 | 62.0 | All | Observational | 33 | Both | Yes |
| Shi et al. [13] | China | 81 | 42.0 | All | Observational | 50 | Both | No |
| Yang et al. [14] | China | 149 | 54.4 | Mild | Observational | 29 | Survivors | Yes |
| Qian et al. [15] | China | 91 | 41 | All | Observational | 27 | Survivors | No |
| Xu et al. [16] | China | 62 | 56.5 | All | Observational | 16 | Both | Yes |
| Deng et al. [17] | China | 33 | 49.0 | All | Observational | 43 | Both | Yes |
| Guan et al. [18] | China | 1099 | 58.1 | All | Observational | 49 | Survivors | Yes |
| Wang et al. [19] | China | 138 | 54.3 | Severe | Observational | 33 | Both | Yes |
| Chang et al. [20] | China | 13 | 77.0 | Mild | Observational | 19 | Survivors | No |
| Wan et al. [21] | China | 135 | 53.3 | Mild | Observational | 16 | Survivors | Yes |
| Young et al. [22] | Singapore | 18 | 50.0 | All | Observational | 33 | Survivors | Yes |
| Wu et al. [23] | China | 201 | | All | Observational | 32 | Survivors | Yes |
| Chan et al. [24] | China | 7 | 50.0 | All | Observational | 14 | Survivors | No |
| Hill et al. [25] | Scotland | 1 | 100 | Mild | Observational | 7 | Both | Yes |
| Jin et al. [26] | China | 651 | 50.8 | All | Observational | 12 | Both | Yes |
| Wang et al [27] | China | 339 | 49.0 | All | Observational | 36 | Both | No |
| Kim et al. [28] | Korea | 1 | 100 | Mild | Observational | 13 | Both | No |
| Lim et al. [29] | Korea | 1 | 50.6 | Mild | Observational | 17 | Survivors | Yes |
| Holshue et al. [30] | USA | 1 | 100 | Mild | Observational | 15 | Survivors | Yes |
| Wang et al. [31] | China | 125 | 56.8 | All | Observational | 29 | Both | Yes |
| Chen et al. [32] | China | 274 | 62.0 | All | Observational | 46 | Both | Yes |
| Zheng et al. [33] | China | 161 | 49.7 | All | Observational | 21 | Both | No |
| Zhao et al. [34] | China | 77 | 44.2 | Mild | Observational | 17 | Both | No |
| Liu et al. [35] | China | 625 | 52.6 | All | Observational | 39 | Both | Yes |
| Li et al. [36] | China | 17 | 53.0 | All | Observational | 19 | Survivors | No |
| Zhang et al. [37] | China | 110 | 68.7 | All | Observational | 52 | Both | Yes |
| Xu [38] | China | 10 | | Severe | Observational | 41 | Both | Yes |
| Zhang et al. [39] | China | 82 | 65.9 | Severe | Observational | 30 | Non-survivors | Yes |
| Miao et al. [40] | China | 163 | 67.3 | All | Observational | 32 | Survivors | Yes |
| Luo et al. [41] | China | 403 | 47.9 | All | Observational | 26 | Both | Yes |
| Shi et al. [42] | China | 101 | 59.4 | Severe | Observational | 45 | Non-survivors | Yes |
| Yao et al. [43] | China | 55 | 67.0 | Severe | Observational | 23 | Non-survivors | Yes |
| Huang et al. [44]. | China | 36 | 69.4 | Severe | Observational | 24 | Non-survivors | Yes |
| Liu et al. [45] | China | 51 | 63.7 | Mild | Observational | 22 | Survivors | Yes |
| Wang et al. [46] | China | 110 | 43.6 | All | Observational | 40 | Both | No |
| Liao et al. [47] | China | 46 | 52.2 | All | Observational | 29 | Survivors | Yes |
| Liu et al. [48] | China | 64 | 36.0 | All | Observational | 39 | Survivors | Yes |
| Qiu et al. [49] | China | 104 | 47.1 | All | Observational | 21 | Survivors | Yes |
| Liao et al. [50] | China | 539 | 63.3 | all | Observational | 59 | Both | Yes |

| | | | | | | | | |
|---|---|---|---|---|---|---|---|---|
| Yan et al. [51] | China | 120 | 45.0 | All | Observational | 38 | Survivors | Yes |
| Tan et al. [52] | China | 2 | 50.0 | Mild | Observational | 40 | Survivors | Yes |
| Zhang et al. [53] | China | 67 | 61.2 | All | Observational | 65 | Both | Yes |
| Jiang et al. [54] | China | 55 | 49.0 | All | Observational | 24 | Both | Yes |
| Qi et al. [55] | China | 21 | 52.4 | All | Observational | 84 | Both | Yes |
| Huang et al. [56] | China | 223 | 46.5 | Mild | Observational | 67 | Both | No |
| Pan et al. [57] | China | 21 | 29.0 | Mild | Observational | 25 | Both | No |
| Xu et al. [58] | China | 51 | 50.0 | Mild | Observational | 26 | Both | No |
| Lillie et al. [59]. | UK | 2 | 50.0 | Mild | Observational | 14 | Both | Yes |
| Ghinai et al. [60] | USA | 2 | 50.0 | Mild | Observational | 26 | Survivors | No |
| Wolfel et al. [61] | Germany | 9 | | All | Observational | 28 | Both | No |
| Ge et al. [62] | China | 7 | 57.0 | All | Observational | 8 | Both | No |
| Xie et al. [63] | China | 79 | 55.7 | All | Observational | 12 | Survivors | Yes |
| Zhou et al. [64] | China | 17 | 64.7 | All | Observational | 16 | Survivors | Yes |
| Duan et al. [65] | China | 10 | 60.0 | Severe | Observational | 28 | Survivors | Yes |
| Zhu et al. [66] | China | 50 | 52.0 | All | Observational | 37 | Both | Yes |
| Sun et al. [67] | China | 83 | 71.1 | All | Observational | 35 | Survivors | No |
| Liu et al. [68] | China | 80 | 42.5 | All | Observational | 26 | Both | Yes |
| Fan et al. [69] | China | 55 | 54.5 | All | Observational | 55 | Survivors | Yes |
| Wang et al. [70] | China | 4 | 75.0 | All | Observational | 15 | Survivors | Yes |
| The COVID-19 Investigation Team [71] | USA | 12 | 67.0 | All | Observational | 33 | Both | No |
| Hu et al. [72] | China | 24 | 33.3 | Mild | Observational | 21 | Survivors | Yes |
| Shen et al. [73] | China | 5 | 60.0 | Severe | Observational | 65 | Both | Yes |
| Auld et al. [74] | USA | 217 | 45.2 | Severe | Observational | 42 | Non-survivors | Yes |
| Tang et al. [75] | China | 183 | 63.4 | Severe | Observational | 34 | Both | No |
| Onder et al. [76] | Italy | 1625 | 28.3 | All | Observational | | Non-survivors | NA |
| Liu et al. [77] | China | 73 | 54.3 | All | Observational | 13 | Both | Yes |
| McMichael et al. [78] | USA | 167 | 32.9 | All | Observational | 20 | Both | No |
| Zhou et al. [79] | Japan | 26 | | Mild | Observational | 40 | Survivors | No |
| Shen et al. [80] | China | 119 | 47.0 | Mild | Observational | 59 | Both | No |
| Lin et al. [81] | China | 95 | 47.4 | All | Observational | 29 | Both | Yes |
| Gudbjartsson et al. [82] | Iceland | 190 | 60.6 | All | Observational | 19 | Both | No |
| Li et al. [83] | China | 86 | 46.5 | All | Randomized | 77 | Survivors | Yes |
| Arons et al. [84] | USA | 48 | | All | Observational | 21 | Both | No |
| COVID-19 Surveillance Group [85] | Italy | 3200 | 70.6 | Severe | Observational | 19 | Non-survivors | No |
| Kang et al. [86] | China | 37 | 48.6 | All | Observational | 26 | Both | No |
| Xu et al. [87] | China | 48 | 27.1 | Severe | Observational | 25 | Non-survivors | Yes |
| Chen et al [88]. | China | 240 | 46.6 | All | Randomized | 21 | Both | Yes |
| Bi et al. [89] | China | 420 | 47.6 | All | Observational | 87 | Both | Yes |
| Lei et al. [90] | China | 308 | 17.4 | All | Observational | 26 | Both | Yes |
| Tian et al. [91] | China | 262 | 48.5 | All | Observational | 21 | Survivors | No |
| Xu et al. [92] | China | 50 | 58.0 | All | Observational | 51 | Both | No |
| Han et al. [93] | China | 206 | 44.2 | All | Observational | 16 | Survivors | No |
| Grasselli et al. [94] | Italy | 4 | 75.0 | Severe | Observational | 36 | Both | Yes |

| | | | | | | | | |
|---|---|---|---|---|---|---|---|---|
| Spinato et al. [95] | UK | 202 | 48.0 | All | Observational | 51 | Both | Yes |
| Chow et al. [96] | USA | 48 | 22.9 | All | Observational | 14 | Both | No |
| Chen et al. [97] | China | 11 | 36.4 | Mild | Observational | 36 | Survivors | Yes |
| Department of Epidemiology [98] | Chile | 922 | 49.0 | All | Observational | 22 | Both | No |
| Li et al. [99] | China | 25 | 40.0 | Severe | Observational | 30 | Non-survivors | No |
| Kluytmans et al. [100] | Netherlands | 86 | 17.4 | All | Observational | | Survivors | No |
| ICARNAC [101] | UK | 165 | 59.3 | All | Observational | 27 | Both | Yes |
| Zhao et al. [102] | China | 173 | 49.0 | All | Observational | 29 | Both | No |
| Petrilli et al. [103] | USA | 1941 | 39.0 | Mild | Observational | 32 | Both | Yes |
| Guo et al. [104] | China | 159 | 62.3 | All | Observational | 68 | Both | Yes |
| Tabata et al [105] | Japan | 104 | 54.0 | All | Observational | 15 | Survivors | Yes |
| Liu et al. [106] | China | 100 | 60.0 | All | Observational | 99 | Both | Yes |
| Tao et al. [107] | China | 167 | | Mild | Observational | 60 | Survivors | No |

**Supplementary Table 2**. Pearson and Spearman analysis (Bonferroni correction) of the blood indices and complications in Covid-19 patients

| | **Liver dys.** | | **Res. fail** | | **Heart fail** | | **Sep. shock** | | **ACI** | | **AKI** | | **Sec. infection** | |
|---|---|---|---|---|---|---|---|---|---|---|---|---|---|---|
| | **Pe** | **Sp** | **Pe** | **Sp** | **Pe** | **Sp** | **Pe** | **Sp** | **Pe** | **Sp** | **Pe** | **Sp** | **Pe** | **Sp** |
| PLT | -0.19 | **0.70** | **-0.75** | **0.99** | **-0.83** | 0.47 | **-0.76** | 0.13 | **-0.85** | 0.23 | -0.61 | -0.14 | -0.42 | 0.40 |
| LYM | 0.01 | -0.11 | -0.41 | -0.41 | -0.64 | -0.18 | -0.42 | -0.56 | -0.42 | -0.51 | -0.28 | -0.03 | -0.39 | -0.43 |
| WBC | 0.29 | **0.68** | 0.52 | **0.99** | **0.77** | 0.47 | 0.64 | 0.13 | 0.57 | 0.23 | 0.46 | -0.13 | 0.39 | 0.41 |
| NEU | 0.20 | 0.62 | 0.55 | **0.99** | 0.71 | 0.38 | 0.50 | 0.37 | 0.60 | 0.07 | 0.64 | -0.23 | **0.76** | 0.40 |
| HBG | -0.13 | 0.66 | -0.37 | **0.99** | -0.44 | 0.73 | -0.32 | 0.37 | -0.37 | 0.24 | -0.34 | -0.13 | -0.30 | 0.40 |
| Albumin | -0.08 | -0.21 | -0.11 | 0.33 | -0.26 | 0.05 | -0.17 | 0.13 | -0.25 | -0.01 | -0.17 | -0.35 | -0.55 | -0.43 |
| ALT | -0.33 | **0.69** | -0.02 | **0.99** | 0.23 | 0.71 | 0.29 | 0.36 | 0.07 | 0.23 | 0.18 | -0.14 | 0.08 | 0.36 |
| AST | 0.32 | 0.66 | 0.37 | **0.99** | 0.56 | 0.72 | 0.26 | 0.36 | 0.18 | 0.23 | 0.48 | -0.14 | 0.64 | 0.40 |
| TB | 0.15 | 0.65 | 0.56 | **0.99** | 0.73 | 0.72 | 0.61 | 0.36 | 0.47 | 0.23 | 0.64 | -0.14 | 0.57 | 0.40 |
| BUN | 0.10 | 0.74 | 0.68 | **0.99** | **0.89** | 0.68 | 0.55 | **0.86** | 0.61 | **0.87** | 0.61 | 0.50 | 0.63 | 0.76 |
| Cr | -0.10 | **0.70** | 0.51 | **0.99** | 0.62 | 0.59 | **0.75** | 0.27 | **0.70** | 0.23 | 0.65 | -0.13 | 0.33 | 0.41 |
| CK | 0.15 | 0.67 | 0.44 | **0.99** | 0.63 | 0.68 | 0.63 | **0.85** | 0.67 | **0.87** | 0.39 | 0.51 | 0.40 | 0.71 |
| CRP | -0.02 | **0.70** | 0.40 | **0.99** | 0.48 | 0.59 | 0.51 | 0.20 | 0.59 | 0.23 | 0.52 | -0.14 | 0.52 | 0.40 |
| GGT | 0.14 | **0.80** | **0.91** | 0.81 | **0.88** | 0.01 | 0.67 | 0.76 | **0.75** | 0.02 | **0.77** | **0.89** | 0.60 | 0.01 |
| IL6 | -0.17 | **0.80** | 0.29 | **0.96** | 0.47 | 0.01 | 0.53 | **0.90** | 0.49 | 0.85 | 0.36 | 0.36 | 0.20 | 0.76 |
| PCT | -0.14 | 0.54 | -0.15 | **0.78** | -0.10 | 0.13 | -0.07 | 0.34 | -0.13 | 0.31 | -0.08 | -0.07 | -0.14 | -0.10 |
| D-dimer | -0.21 | 0.57 | -0.15 | **0.80** | -0.09 | 0.27 | -0.08 | 0.13 | -0.15 | -0.06 | -0.16 | -0.43 | -0.13 | -0.15 |

Pe: Pearson correlation, Sp: Spearman correlation; dys: dysfunction; Res: respiratory; fail: failure; Sep: septic; ACI: acute cardiac injury; AKI: acute kidney injury; TB: total bilirubin; CK: Creatine Kinase; Sec: secondary; Cr: creatinine; Bold values denote statistical significance at the $P < 0.05$.

**Supplementary Table 3**. Pearson and Spearman analysis (Bonferroni correction) of the pre-existing conditions and complications in Covid-19 patients

| | Liver dys. | | Res. fail | | Heart fail | | Sep. shock | | ACI | | AKI | | Sec. infection | |
|---|---|---|---|---|---|---|---|---|---|---|---|---|---|---|
| | **Pe** | **Sp** | **Pe** | **Sp** | **Pe** | **Sp** | **Pe** | **Sp** | **Pe** | **Sp** | **Pe** | **Sp** | **Pe** | **Sp** |
| Age | 0.14 | 0.04 | 0.69 | **0.77** | **0.80** | 0.72 | 0.66 | **0.70** | 0.66 | **0.75** | 0.63 | 0.53 | 0.50 | 0.67 |
| Male | 0.16 | -0.13 | 0.43 | -0.04 | 0.45 | 0.21 | 0.49 | -0.14 | 0.61 | 0.25 | 0.43 | 0.17 | 0.49 | 0.06 |
| Smoking | 0.40 | 0.27 | 0.24 | 0.29 | 0.32 | 0.29 | 0.34 | 0.25 | 0.28 | 0.24 | 0.17 | 0.23 | 0.20 | 0.27 |
| Comorb. | 0.09 | 0.33 | 0.64 | **0.74** | **0.78** | **0.82** | 0.62 | **0.73** | 0.69 | 0.66 | 0.56 | 0.69 | 0.39 | **0.83** |
| Hyperten. | 0.18 | -0.01 | 0.21 | 0.63 | 0.51 | 0.66 | 0.23 | **0.70** | 0.25 | 0.61 | 0.26 | 0.54 | 0.36 | 0.70 |
| Diabetes | -0.30 | 0.24 | 0.39 | **0.69** | 0.48 | **0.81** | 0.49 | **0.77** | 0.52 | 0.66 | 0.45 | 0.54 | 0.33 | **0.70** |
| Cardiovas. | 0.41 | 0.33 | 0.46 | 0.66 | **0.79** | **0.71** | 0.28 | **0.69** | 0.23 | **0.70** | 0.33 | **0.68** | 0.57 | **0.88** |
| COPD | -0.24 | 0.52 | 0.38 | 0.67 | 0.57 | 0.70 | 0.41 | 0.36 | 0.49 | **0.78** | 0.41 | 0.57 | 0.35 | 0.69 |
| Cancer | -0.25 | 0.51 | 0.15 | 0.49 | 0.19 | 0.54 | -0.07 | 0.36 | 0.05 | 0.26 | 0.20 | -0.01 | 0.27 | 0.39 |
| Liver D. | -0.25 | 0.20 | -0.03 | 0.23 | 0.32 | 0.19 | 0.47 | 0.33 | 0.24 | 0.31 | 0.25 | 0.56 | 0.09 | 0.21 |
| Cerebrov. | -0.16 | 0.14 | 0.58 | 0.59 | **0.75** | **0.88** | **0.73** | 0.70 | 0.67 | 0.72 | 0.55 | 0.84 | 0.33 | **0.77** |
| Renal D. | 0.04 | 0.25 | 0.34 | 0.64 | 0.43 | **0.72** | -0.01 | 0.56 | 0.16 | **0.72** | 0.39 | **0.70** | 0.59 | 0.38 |
| Other | -0.08 | 0.56 | 0.34 | 0.52 | 0.19 | 0.20 | 0.40 | 0.20 | 0.52 | 0.45 | 0.28 | 0.40 | -0.09 | 0.46 |

Pe: Pearson correlation, Sp: Spearman correlation; dys: dysfunction; Res: respiratory; fail: failure; Sep: septic; ACI: acute cardiac injury; AKI: acute kidney injury; Sec: secondary; Comorb: comorbidities; D: disease; Hyperten: hypertension; Cardiovas: cardiovascular disease; Cerebrov: cerebrovascular disease. Bold values denote statistical significance at the $P < 0.05$.

**Supplementary Table** 4. Multicollinearity is measured using Variance Inflation Factors (VIF) withinblood indices associated with complications

**Multicollinearity is measured using Variance Inflation Factors (VIF) withinblood indices associated with complications**

**Secondary infection**

| Statistic | PLT | LYM | WBC | NEU | HBG | Albumin | ALT | AST | TB | BUN | Creatinine | CK | CRP | GGT | IL6 | PCT | D-Dimer |
|---|---|---|---|---|---|---|---|---|---|---|---|---|---|---|---|---|---|
| $R^2$ | 0.99 | 0.84 | 0.98 | 0.96 | 0.86 | 0.88 | 0.92 | 0.94 | 0.99 | 0.95 | 0.98 | 0.98 | 0.94 | 0.99 | 0.97 | 0.92 | 0.75 |
| Tolerance | 0.01 | 0.16 | 0.02 | 0.04 | 0.14 | 0.12 | 0.08 | 0.06 | 0.01 | 0.05 | 0.02 | 0.02 | 0.06 | 0.01 | 0.03 | 0.08 | 0.25 |
| VIF | 152.85 | 6.19 | 49.05 | 23.72 | 7.25 | 8.41 | 12.44 | 17.49 | 71.32 | 21.06 | 45.28 | 49.08 | 16.10 | 74.69 | 39.99 | 13.26 | 4.05 |

**Acute kidney injury**

| Statistic | PLT | LYM | WBC | NEU | HBG | Albumin | ALT | AST | TB | BUN | Creatinine | CK | CRP | GGT | IL6 | PCT | D-Dimer |
|---|---|---|---|---|---|---|---|---|---|---|---|---|---|---|---|---|---|
| $R^2$ | 0.99 | 0.84 | 0.98 | 0.95 | 0.85 | 0.88 | 0.90 | 0.91 | 0.99 | 0.96 | 0.97 | 0.98 | 0.94 | 0.99 | 0.97 | 0.93 | 0.75 |
| Tolerance | 0.01 | 0.16 | 0.02 | 0.05 | 0.15 | 0.12 | 0.10 | 0.09 | 0.01 | 0.04 | 0.03 | 0.02 | 0.06 | 0.01 | 0.03 | 0.07 | 0.25 |
| VIF | 150.93 | 6.19 | 41.87 | 20.87 | 6.81 | 8.55 | 10.08 | 11.37 | 68.52 | 26.27 | 39.70 | 48.53 | 18.02 | 86.10 | 36.95 | 13.48 | 3.97 |

**Acute cardiac injury**

| Statistic | PLT | LYM | WBC | NEU | HBG | Albumin | ALT | AST | TB | BUN | Creatinine | CK | CRP | GGT | IL6 | PCT | D-Dimer |
|---|---|---|---|---|---|---|---|---|---|---|---|---|---|---|---|---|---|
| $R^2$ | 1.00 | 0.84 | 0.98 | 0.98 | 0.96 | 0.91 | 0.91 | 0.90 | 0.99 | 0.96 | 0.98 | 0.99 | 0.97 | 0.99 | 0.98 | 0.96 | 0.87 |
| Tolerance | 0.00 | 0.16 | 0.02 | 0.02 | 0.04 | 0.09 | 0.09 | 0.10 | 0.01 | 0.04 | 0.02 | 0.01 | 0.03 | 0.01 | 0.02 | 0.04 | 0.13 |
| VIF | 290.63 | 6.13 | 42.59 | 42.14 | 22.49 | 11.57 | 11.41 | 10.50 | 70.61 | 24.10 | 47.80 | 72.18 | 34.48 | 73.12 | 52.73 | 22.39 | 7.83 |

**Septic shock**

| Statistic | PLT | LYM | WBC | NEU | HBG | Albumin | ALT | AST | TB | BUN | Creatinine | CK | CRP | GGT | IL6 | PCT | D-Dimer |
|---|---|---|---|---|---|---|---|---|---|---|---|---|---|---|---|---|---|
| $R^2$ | 1.00 | 0.99 | 0.99 | 1.00 | 1.00 | 1.00 | 0.99 | 1.00 | 1.00 | 1.00 | 1.00 | 1.00 | 0.99 | 1.00 | 1.00 | 1.00 | 1.00 |
| Tolerance | 0.00 | 0.01 | 0.01 | 0.00 | 0.00 | 0.00 | 0.01 | 0.00 | 0.00 | 0.00 | 0.00 | 0.00 | 0.01 | 0.00 | 0.00 | 0.00 | 0.00 |
| VIF | 20271.19 | 70.58 | 85.44 | 11544.65 | 3167.00 | 3634.56 | 125.67 | 314.27 | 1919.01 | 809.94 | 4216.83 | 3449.87 | 179.02 | 2288.25 | 1087.42 | 3032.20 | 874.99 |

**Heart failure**

| Statistic | PLT | LYM | WBC | NEU | HBG | Albumin | ALT | AST | TB | BUN | Creatinine | CK | CRP | GGT | IL6 | PCT | D-Dimer |
|---|---|---|---|---|---|---|---|---|---|---|---|---|---|---|---|---|---|
| $R^2$ | 0.99 | 0.91 | 0.99 | 0.96 | 0.86 | 0.97 | 0.95 | 0.91 | 0.99 | 0.99 | 0.99 | 0.99 | 0.99 | 0.99 | 0.99 | 0.93 | 0.75 |
| Tolerance | 0.01 | 0.09 | 0.01 | 0.04 | 0.14 | 0.03 | 0.05 | 0.09 | 0.01 | 0.01 | 0.01 | 0.01 | 0.01 | 0.01 | 0.01 | 0.07 | 0.25 |
| VIF | 184.19 | 11.06 | 79.61 | 26.94 | 6.99 | 30.16 | 21.72 | 11.24 | 103.74 | 112.89 | 100.21 | 90.45 | 81.06 | 103.94 | 67.49 | 13.70 | 4.08 |

**Respiratory failure**

| Statistic | PLT | LYM | WBC | NEU | HBG | Albumin | ALT | AST | TB | BUN | Creatinine | CK | CRP | GGT | IL6 | PCT | D-Dimer |
|---|---|---|---|---|---|---|---|---|---|---|---|---|---|---|---|---|---|
| $R^2$ | 1.00 | 0.88 | 0.98 | 0.95 | 0.91 | 0.95 | 0.90 | 0.97 | 0.99 | 0.99 | 0.99 | 0.99 | 0.98 | 1.00 | 0.98 | 0.92 | 0.78 |
| Tolerance | 0.00 | 0.12 | 0.02 | 0.05 | 0.09 | 0.05 | 0.10 | 0.03 | 0.01 | 0.01 | 0.01 | 0.01 | 0.02 | 0.00 | 0.02 | 0.08 | 0.22 |
| VIF | 213.17 | 8.06 | 43.45 | 21.00 | 11.06 | 20.66 | 10.36 | 31.81 | 68.49 | 145.84 | 69.36 | 69.87 | 52.05 | 234.49 | 42.11 | 13.07 | 4.48 |

**Liver dysfunction**

| Statistic | PLT | LYM | WBC | NEU | HBG | Albumin | ALT | AST | TB | BUN | Creatinine | CK | CRP | GGT | IL6 | PCT | D-Dimer |
|---|---|---|---|---|---|---|---|---|---|---|---|---|---|---|---|---|---|
| $R^2$ | 0.99 | 0.84 | 0.98 | 0.96 | 0.87 | 0.91 | 0.90 | 0.92 | 0.99 | 0.97 | 0.98 | 0.98 | 0.94 | 0.99 | 0.97 | 0.93 | 0.75 |
| Tolerance | 0.01 | 0.16 | 0.02 | 0.04 | 0.13 | 0.09 | 0.10 | 0.08 | 0.01 | 0.03 | 0.02 | 0.02 | 0.06 | 0.01 | 0.03 | 0.07 | 0.25 |
| VIF | 151.16 | 6.13 | 43.50 | 26.87 | 7.70 | 10.76 | 10.44 | 13.17 | 69.07 | 29.90 | 45.16 | 49.04 | 16.06 | 73.95 | 36.73 | 14.32 | 3.98 |

Variance inflation factor measures how much the behavior (variance) of an independent variable is influenced, or inflated, by its interaction/correlation with the other independent variables. Variance inflation factors allow a quick measure of how much a variable is contributing to the standard error in the regression. If the VIF is equal to

1 there is no multicollinearity among factors, but if the VIF is greater than 1, the predictors may be moderately correlated, but not enough to be overly concerned about. A VIF between 5 and 10 indicates high correlation that may be problematic. And if the VIF goes above 10, you can assume that the regression coefficients are poorly estimated due to multicollinearity.

**Supplementary Table 5**. Multicollinearity is measured using Variance Inflation Factors (VIF) within pre-existing health conditions associated with complications

**Multicollinearity is measured using Variance Inflation Factors (VIF) within pre-existing health conditions associated with complications**

**Liver dysfunction**

| Statistic | Age | Male | Smoking | Any Comorbidity | Hypertension | Diabetes | Cardiovascular D. | COPD | Cancer | Liver D. | Cerebrovascular D. | Renal D. | Other |
|---|---|---|---|---|---|---|---|---|---|---|---|---|---|
| $R^2$ | 0.75 | 0.56 | 0.88 | 0.82 | 0.72 | 0.87 | 0.84 | 0.93 | 0.63 | 0.40 | 0.93 | 0.95 | 0.55 |
| Tolerance | 0.25 | 0.44 | 0.12 | 0.18 | 0.28 | 0.13 | 0.16 | 0.07 | 0.37 | 0.60 | 0.07 | 0.05 | 0.45 |
| VIF | 3.92 | 2.29 | 8.33 | 5.57 | 3.58 | 7.88 | 6.44 | 14.49 | 2.72 | 1.66 | 14.35 | 19.01 | 2.23 |

**Respiratory failure**

| Statistic | Age | Male | Smoking | Any Comorbidity | Hypertension | Diabetes | Cardiovascular D. | COPD | Cancer | Liver D. | Cerebrovascular D. | Renal D. | Other |
|---|---|---|---|---|---|---|---|---|---|---|---|---|---|
| $R^2$ | 0.75 | 0.54 | 0.78 | 0.86 | 0.75 | 0.82 | 0.83 | 0.93 | 0.60 | 0.46 | 0.94 | 0.92 | 0.56 |
| Tolerance | 0.25 | 0.46 | 0.22 | 0.14 | 0.25 | 0.18 | 0.17 | 0.07 | 0.40 | 0.54 | 0.06 | 0.08 | 0.44 |
| VIF | 3.94 | 2.17 | 4.63 | 6.91 | 3.98 | 5.50 | 5.90 | 14.60 | 2.49 | 1.86 | 16.88 | 12.16 | 2.26 |

**Heart failure**

| Statistic | Age | Male | Smoking | Any Comorbidity | Hypertension | Diabetes | Cardiovascular D. | COPD | Cancer | Liver D. | Cerebrovascular D. | Renal D. | Other |
|---|---|---|---|---|---|---|---|---|---|---|---|---|---|
| $R^2$ | 0.88 | 0.72 | 0.77 | 0.91 | 0.78 | 0.86 | 0.95 | 0.91 | 0.63 | 0.52 | 0.93 | 0.92 | 0.67 |
| Tolerance | 0.12 | 0.28 | 0.23 | 0.09 | 0.22 | 0.14 | 0.05 | 0.09 | 0.37 | 0.48 | 0.07 | 0.08 | 0.33 |
| VIF | 8.62 | 3.58 | 4.28 | 11.29 | 4.59 | 7.40 | 20.95 | 10.59 | 2.73 | 2.09 | 14.05 | 11.80 | 3.03 |

**Septic shock**

| Statistic | Age | Male | Smoking | Any Comorbidity | Hypertension | Diabetes | Cardiovascular D. | COPD | Cancer | Liver D. | Cerebrovascular D. | Renal D. | Other |
|---|---|---|---|---|---|---|---|---|---|---|---|---|---|
| $R^2$ | 0.87 | 0.79 | 0.82 | 0.92 | 0.84 | 0.89 | 0.86 | 0.92 | 0.61 | 0.61 | 0.94 | 0.97 | 0.59 |
| Tolerance | 0.13 | 0.21 | 0.18 | 0.08 | 0.16 | 0.11 | 0.14 | 0.08 | 0.39 | 0.39 | 0.06 | 0.03 | 0.41 |
| VIF | 8.00 | 4.79 | 5.53 | 11.78 | 6.45 | 9.22 | 7.40 | 12.14 | 2.55 | 2.58 | 17.63 | 29.10 | 2.43 |

**Acute cardiac injury**

| Statistic | Age | Male | Smoking | Any Comorbidity | Hypertension | Diabetes | Cardiovascular D. | COPD | Cancer | Liver D. | Cerebrovascular D. | Renal D. | Other |
|---|---|---|---|---|---|---|---|---|---|---|---|---|---|
| $R^2$ | 0.77 | 0.72 | 0.76 | 0.88 | 0.75 | 0.84 | 0.83 | 0.92 | 0.63 | 0.36 | 0.93 | 0.93 | 0.58 |
| Tolerance | 0.23 | 0.28 | 0.24 | 0.12 | 0.25 | 0.16 | 0.17 | 0.08 | 0.37 | 0.64 | 0.07 | 0.07 | 0.42 |
| VIF | 4.31 | 3.55 | 4.24 | 8.43 | 3.93 | 6.32 | 5.98 | 11.84 | 2.67 | 1.55 | 15.11 | 15.10 | 2.38 |

**Acute kidney injury**

| Statistic | Age | Male | Smoking | Any Comorbidity | Hypertension | Diabetes | Cardiovascular D. | COPD | Cancer | Liver D. | Cerebrovascular D. | Renal D. | Other |
|---|---|---|---|---|---|---|---|---|---|---|---|---|---|
| $R^2$ | 0.74 | 0.54 | 0.78 | 0.80 | 0.71 | 0.82 | 0.84 | 0.92 | 0.60 | 0.36 | 0.93 | 0.92 | 0.55 |
| Tolerance | 0.26 | 0.46 | 0.22 | 0.20 | 0.29 | 0.18 | 0.16 | 0.08 | 0.40 | 0.64 | 0.07 | 0.08 | 0.45 |
| VIF | 3.81 | 2.17 | 4.60 | 4.89 | 3.45 | 5.63 | 6.35 | 12.26 | 2.50 | 1.57 | 15.16 | 12.62 | 2.24 |

**Secondary infection**

| Statistic | Age | Male | Smoking | Any Comorbidity | Hypertension | Diabetes | Cardiovascular D. | COPD | Cancer | Liver D. | Cerebrovascular D. | Renal D. | Other |
|---|---|---|---|---|---|---|---|---|---|---|---|---|---|
| $R^2$ | 0.73 | 0.72 | 0.82 | 0.79 | 0.71 | 0.82 | 0.83 | 0.93 | 0.59 | 0.35 | 0.93 | 0.95 | 0.77 |
| Tolerance | 0.27 | 0.28 | 0.18 | 0.21 | 0.29 | 0.18 | 0.17 | 0.07 | 0.41 | 0.65 | 0.07 | 0.05 | 0.23 |
| VIF | 3.75 | 3.63 | 5.55 | 4.84 | 3.45 | 5.45 | 5.80 | 13.90 | 2.46 | 1.55 | 14.92 | 20.03 | 4.36 |

Variance inflation factor measures how much the behavior (variance) of an independent variable is influenced, or inflated, by its interaction/correlation with the other independent variables. Variance inflation factors allow a quick measure of how much a variable is contributing to the standard error in the regression. If the VIF is equal to

1 there is no multicollinearity among factors, but if the VIF is greater than 1, the predictors may be moderately correlated, but not enough to be overly concerned about. A VIF between 5 and 10 indicates high correlation that may be problematic. And if the VIF goes above 10, you can assume that the regression coefficients are poorly estimated due to multicollinearity. D: disease, COPD: Chronic obstructive pulmonary.

**Supplementary Table 6.** Risk of bias according to the QUIPS tool (+: High risk of bias, +/−: moderate risk of bias, -: low risk of bias, NA: not applicable).

| **Study** | **Participation** | **Attrition** | **Prognostic Factor** | **Outcome** | **Statistical Analysis and Reporting** | **Study Confounding** | **Risk of bias: + = high, +/− = moderate, - = low** |
|---|---|---|---|---|---|---|---|
| Yang et al. [4] | Moderate | Low | Low | Low | High | High | +/− |
| Zhou et al. [12] | Low | Moderate | Low | Low | Low | Low | - |
| Shi et al. [13] | Moderate | Low | Moderate | moderate | Moderate | Moderate | +/− |
| Yang et al. [14] | Low | Low | Low | High | High | High | +/- |
| Qian et al. [15] | Moderate | Low | Low | Moderate | Moderate | High | +/- |
| Xu et al. [16] | High | High | Low | Low | High | Moderate | + |
| Deng et al. [17] | High | Low | Moderate | Low | Moderate | Moderate | +/- |
| Guan et al. [18] | Low | Low | Low | Low | High | High | +/- |
| Wang et al. [19] | Moderate | Low | Low | Low | Moderate | Moderate | +/- |
| Chang et al. [20] | High | Low | Low | Low | High | High | +/- |
| Wan et al. [21] | Moderate | Low | Low | Low | Moderate | Moderate | +/- |
| Young et al. [22] | High | Low | Low | Low | High | High | +/- |
| Wu et al. [23] | Moderate | Low | Low | Low | Low | Low | - |
| Chan et al. [24] | NA | Low | Low | Low | NA | NA | - |
| Hill et al. [25] | High | Low | Moderate | Low | NA | NA | +/- |
| Jin et al. [26] | Low | Low | Low | Low | Low | Low | - |
| Wang et al [27] | Low | Low | Low | Low | Low | Low | - |
| Kim et al. [28] | NA | Low | High | Low | NA | NA | +/- |
| Lim et al. [29] | High | Low | High | Low | NA | NA | +/- |
| Holshue et al. [30] | NA | Low | Low | Low | NA | NA | - |
| Wang et al. [31] | Moderate | Low | Low | Low | Moderate | Moderate | +/- |
| Chen et al. [32] | Low | Low | Low | Low | Low | Moderate | - |
| Zheng et al. [33] | Low | High | Low | Moderate | High | High | +/- |
| Zhao et al. [34] | Moderate | Low | Low | Low | Low | Low | - |
| Liu et al. [35] | Low | Low | Low | Moderate | Low | Low | - |
| Li et al. [36] | High | Low | Low | Moderate | High | High | +/- |
| Zhang et al. [37] | Moderate | Low | Low | Low | Low | Low | - |
| Xu [38] | High | High | Low | Low | NA | NA | +/- |
| Zhang et al. [39] | Moderate | Low | Low | Low | Moderate | Moderate | +/- |
| Miao et al. [40] | Low | High | Low | Moderate | Moderate | High | +/- |
| Luo et al. [41] | Low | Moderate | Low | Low | Moderate | High | +/- |
| Shi et al. [42] | Moderate | Moderate | Low | Low | Low | High | +/- |
| Yao et al. [43] | Moderate | Low | Low | Low | Moderate | High | +/- |
| Huang et al. [44]. | Moderate | Low | Low | Low | High | High | +/- |
| Liu et al. [45] | High | High | Low | Low | Moderate | High | +/- |
| Wang et al. [46] | Moderate | High | Moderate | Low | Low | Moderate | +/- |
| Liao et al. [47] | High | High | Low | Low | Low | Moderate | +/- |
| Liu et al. [48] | Moderate | Moderate | Low | Low | Low | Low | - |
| Qiu et al. [49] | Moderate | Low | Low | Low | Moderate | Moderate | +/- |
| Liao et al. [50] | High | Low | Low | Low | Moderate | Moderate | +/- |

| | | | | | | | |
|---|---|---|---|---|---|---|---|
| Yan et al. [51] | Moderate | High | Low | Low | Low | Low | - |
| Tan et al. [52] | High | Moderate | Moderate | Moderate | High | High | + |
| Zhang et al. [53] | Moderate | Low | Low | Low | High | High | +/- |
| Jiang et al. [54] | High | Low | Low | Low | Moderate | High | +/- |
| Qi et al. [55] | High | Low | Low | Low | High | High | +/- |
| Huang et al. [56] | Low | High | Low | High | High | High | +/- |
| Pan et al. [57] | High | Low | Low | Moderate | Moderate | High | +/- |
| Xu et al. [58] | High | Low | Low | Low | High | High | +/- |
| Lillie et al. [59]. | NA | Low | Moderate | High | NA | NA | +/- |
| Ghinai et al. [60] | NA | Low | Moderate | Moderate | NA | NA | +/- |
| Wolfel et al. [61] | High | NA | Low | Low | Low | Low | - |
| Ge et al. [62] | NA | Low | Moderate | High | NA | NA | +/- |
| Xie et al. [63] | Moderate | High | Low | Low | Low | High | +/- |
| Zhou et al. [64] | High | High | Low | High | Low | High | + |
| Duan et al. [65] | Moderate | Low | Low | Low | High | High | +/- |
| Zhu et al. [66] | Low | Moderate | Low | Low | High | High | +/- |
| Sun et al. [67] | High | Moderate | Low | Low | Low | Low | - |
| Liu et al. [68] | High | Low | Low | Low | Moderate | High | +/- |
| Fan et al. [69] | High | Low | Low | Low | High | High | +/- |
| Wang et al. [70] | NA | Low | Low | Low | NA | NA | - |
| The COVID-19 Investigation Team [71] | NA | Low | Low | Moderate | NA | NA | - |
| Hu et al. [72] | High | Low | Moderate | Low | High | High | +/- |
| Shen et al. [73] | High | Low | Low | Low | NA | NA | - |
| Auld et al. [74] | Low | High | Moderate | Low | Moderate | High | +/- |
| Tang et al. [75] | Moderate | Moderate | Low | Low | Moderate | High | +/- |
| Onder et al. [76] | Low | High | Moderate | Moderate | NA | NA | +/- |
| Liu et al. [77] | High | Low | Moderate | Low | High | High | +/- |
| McMichael et al. [78] | Moderate | Moderate | Moderate | Low | NA | NA | +/- |
| Zhou et al. [79] | High | NA | NA | Low | NA | NA | +/- |
| Shen et al. [80] | High | High | Low | Moderate | High | High | + |
| Lin et al. [81] | Moderate | Low | Low | Low | High | High | +/- |
| Gudbjartsson et al. [82] | Low | Low | Low | Low | Low | Low | - |
| Li et al. [83] | Moderate | Low | Low | Low | High | High | +/- |
| Arons et al. [84] | Moderate | Low | Moderate | Low | High | High | +/- |
| COVID-19 Surveillance Group [85] | Low | NA | Low | Low | NA | NA | - |
| Kang et al. [86] | Low | High | Moderate | Moderate | High | High | + |
| Xu et al. [87] | High | High | Low | Moderate | High | High | +/- |
| Chen et al. [88]. | Low | Low | Low | Low | Moderate | High | +/- |
| Bi et al. [89] | Moderate | Low | Low | Low | Low | Low | - |
| Lei et al. [90] | Moderate | Moderate | Low | Low | Moderate | High | +/- |
| Tian et al. [91] | Low | Low | Moderate | Low | High | High | +/- |
| Xu et al. [92] | High | Low | Low | Low | High | High | +/- |

| | | | | | | | |
|---|---|---|---|---|---|---|---|
| Han et al. [93] | Moderate | Low | Low | Low | High | High | +/- |
| Grasselli et al. [94] | Low | Low | Moderate | Low | Moderate | Moderate | +/- |
| Spinato et al. [95] | Moderate | Low | High | Moderate | High | High | +/- |
| Chow et al. [96] | Moderate | High | High | Low | NA | NA | +/- |
| Chen et al. [97] | NA | High | Low | Low | High | High | +/- |
| Department of Epidemiology [98] | Low | High | Low | Low | High | High | +/- |
| Li et al. [99] | High | NA | Low | Low | High | High | +/- |
| Kluytmans et al. [100] | Moderate | Low | Low | Low | NA | NA | - |
| ICARNAC [101] | Low | Low | Low | Low | NA | NA | - |
| Zhao et al. [102] | Moderate | Low | Low | Low | Low | High | +/- |
| Petrilli et al. [103] | Low | Low | Low | Low | Low | Low | - |
| Guo et al. [104] | Moderate | Low | Moderate | Low | High | High | +/- |
| Tabata et al [105] | Moderate | Low | Low | Low | High | High | +/- |
| Liu et al. [106] | Low | Moderate | Moderate | Low | Low | Low | +/- |
| Tao et al. [107] | High | High | Low | High | High | High | + |
| Overall: High risk | 30/97 | 20/97 | 4/97 | 6/97 | 35/97 | 50/97 | 6/97 |
| Overall: moderate risk | 34/97 | 11/97 | 19/97 | 15/97 | 21/97 | 13/97 | 68/97 |
| Overall: Low risk | 24/97 | 62/97 | 73/97 | 76/97 | 22/97 | 15/97 | 23/97 |

In the case of participation, the study was judged to have a low risk of bias if the diagnosis of mortality for COVID-19 patients was explicitly specified, with adequate participation in the study by individuals. In the case of study attrition, a good explanation of patient health follow-up indicated a low risk of bias, a moderate risk of bias indicated an incomplete or short follow-up, and a high risk of bias indicated a lack of description. In terms of the outcome, a clear description of the outcomes was needed to achieve low risk of bias. Among SARS-Cov-2 patients, if outcomes were specifically and separately reported, a low risk of bias was achieved. If multipurpose factors related to COVID-19 infection were assessed, a sufficient prognostic factor with a low risk of bias on an individual study basis. In the case of statistical analysis and confounding factors, the rater attributed a low risk of bias to the study provided enough information available to explain the statistical methods used. The use of suitable statistical method, the establishment of a multivariate analysis indicates, and considerations of major confounding factors (e.g. age, gender, treatment etc.) suggested a low risk of bias.
