# Supplementary material for: Risk of mortality in COVID-19 patients: a meta- and network analysis
Source: Sci Rep. 2023 Feb 6;13:2138. doi: 10.1038/s41598-023-29364-8 (PMC9901837; doi:10.1038/s41598-023-29364-8)

Meta-analysis of blood parameters in survivors and non-survivors of COVID-19

Table.1 summary of studies

	Study	N1	Mean1	Sd1	N2	Mean2	Sd2	Moderator	subgroup	Cohen's d	CorrectionFactor	Hedges'g (SMD)	SEg	95%CI-Lower	95%CI-Upper	weight(%)-fixed model	weight(%)-random model %
1	GGT	1582	11.06	10.02	60	52.50	12.02	0.0	NAN	4.103449	0.999543	4.101572	0.149685	3.808189	4.394955	0.634392	5.834679
2	NEU	4237	3.55	0.91	604	6.48	1.71	0.0	NAN	2.807348	0.999845	2.806913	0.052008	2.704978	2.908848	5.255063	5.884626
3	Creatinine	4670	66.04	6.86	636	87.52	18.03	0.0	NAN	2.396382	0.999859	2.396043	0.048238	2.301495	2.490590	6.108413	5.885592
4	WBC	6591	5.39	1.06	583	8.55	3.00	0.0	NAN	2.380073	0.999895	2.379824	0.047553	2.286620	2.473029	6.285674	5.885760
5	CRP	4297	23.11	27.60	598	96.39	54.49	0.0	NAN	2.282116	0.999847	2.281766	0.049358	2.185025	2.378508	5.834500	5.885313
6	AST	4526	30.18	12.32	494	50.68	25.79	0.0	NAN	1.441603	0.999851	1.441388	0.049513	1.344343	1.538432	5.798070	5.885273
7	CK	3706	73.20	20.99	338	101.00	47.85	0.0	NAN	1.139935	0.999814	1.139723	0.058205	1.025642	1.253805	4.195607	5.882880
8	IL-6	1557	18.96	43.00	270	59.67	41.40	0.0	NAN	0.951882	0.999589	0.951490	0.067751	0.818699	1.084282	3.096617	5.879809
9	BUN	3326	5.66	6.47	475	8.55	1.76	0.0	NAN	0.474956	0.999803	0.474863	0.049342	0.378152	0.571573	5.838224	5.885316
10	Bilirubin	4206	13.41	10.67	552	15.47	5.41	0.0	NAN	0.201951	0.999842	0.201919	0.045310	0.113112	0.290727	6.923547	5.886293
11	ALT	4705	29.93	17.70	548	30.09	9.99	0.0	NAN	0.009379	0.999857	0.009377	0.045131	-0.079079	0.097833	6.978629	5.886334
12	D-Dimer	3390	7.44	29.74	663	5.81	4.66	0.0	NAN	-0.059780	0.999815	-0.059769	0.042462	-0.142995	0.023458	7.883269	5.886932
13	PCT	3374	0.83	3.94	638	0.41	0.34	0.0	NAN	-0.116148	0.999813	-0.116126	0.043183	-0.200765	-0.031488	7.622383	5.886774
14	Albumin	3040	38.51	8.82	451	31.93	2.13	0.0	NAN	-0.795931	0.999785	-0.795760	0.051340	-0.896387	-0.695132	5.392567	5.884802
15	HBG	5887	134.64	8.50	492	124.03	7.42	0.0	NAN	-1.259831	0.999882	-1.259682	0.048231	-1.354215	-1.165149	6.110259	5.885594
16	PLT	6572	187.77	23.39	690	149.92	32.55	0.0	NAN	-1.550759	0.999897	-1.550598	0.042032	-1.632980	-1.468217	8.045718	5.887025
17	LYM	6943	1.17	0.34	697	0.60	0.14	0.0	NAN	-1.743746	0.999902	-1.743575	0.042159	-1.826207	-1.660943	7.997067	5.886998

Figure.1 Forestplot - fixed and random effect models

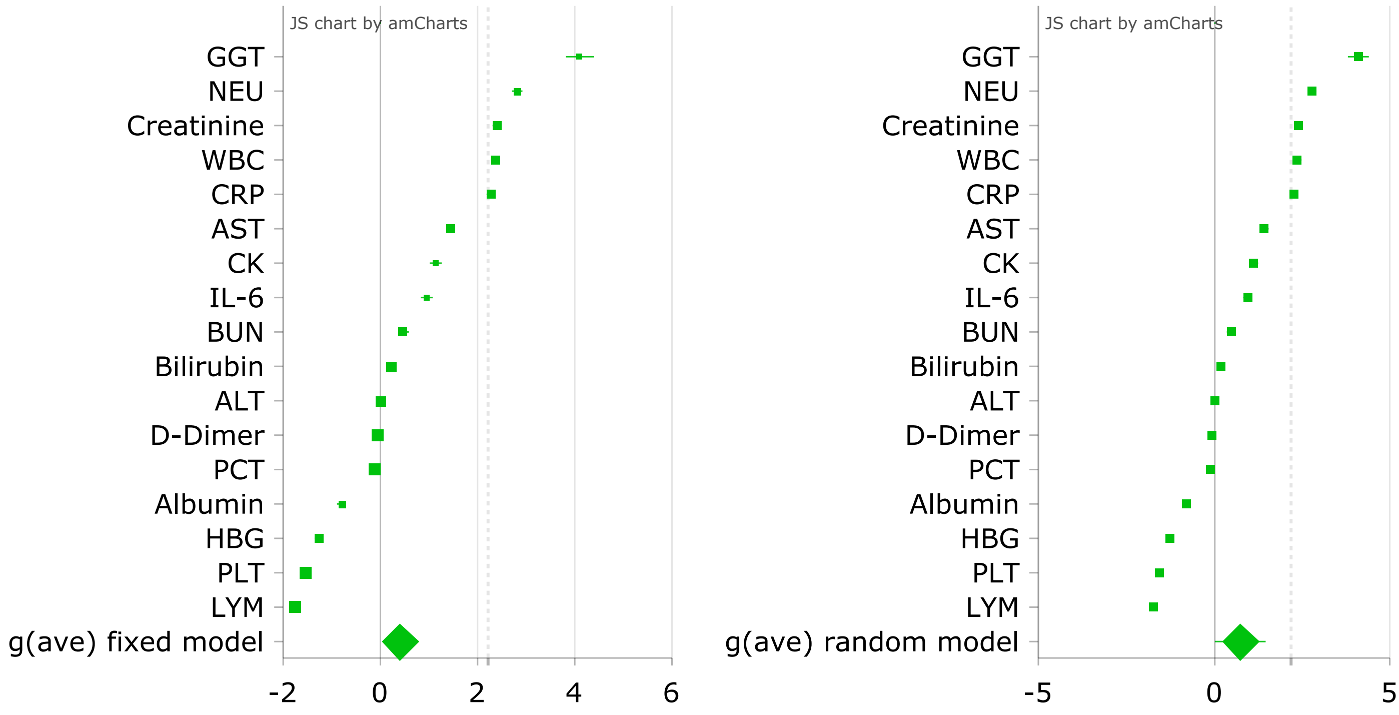

Figure.2 Funnel Plot - fixed and random effect models

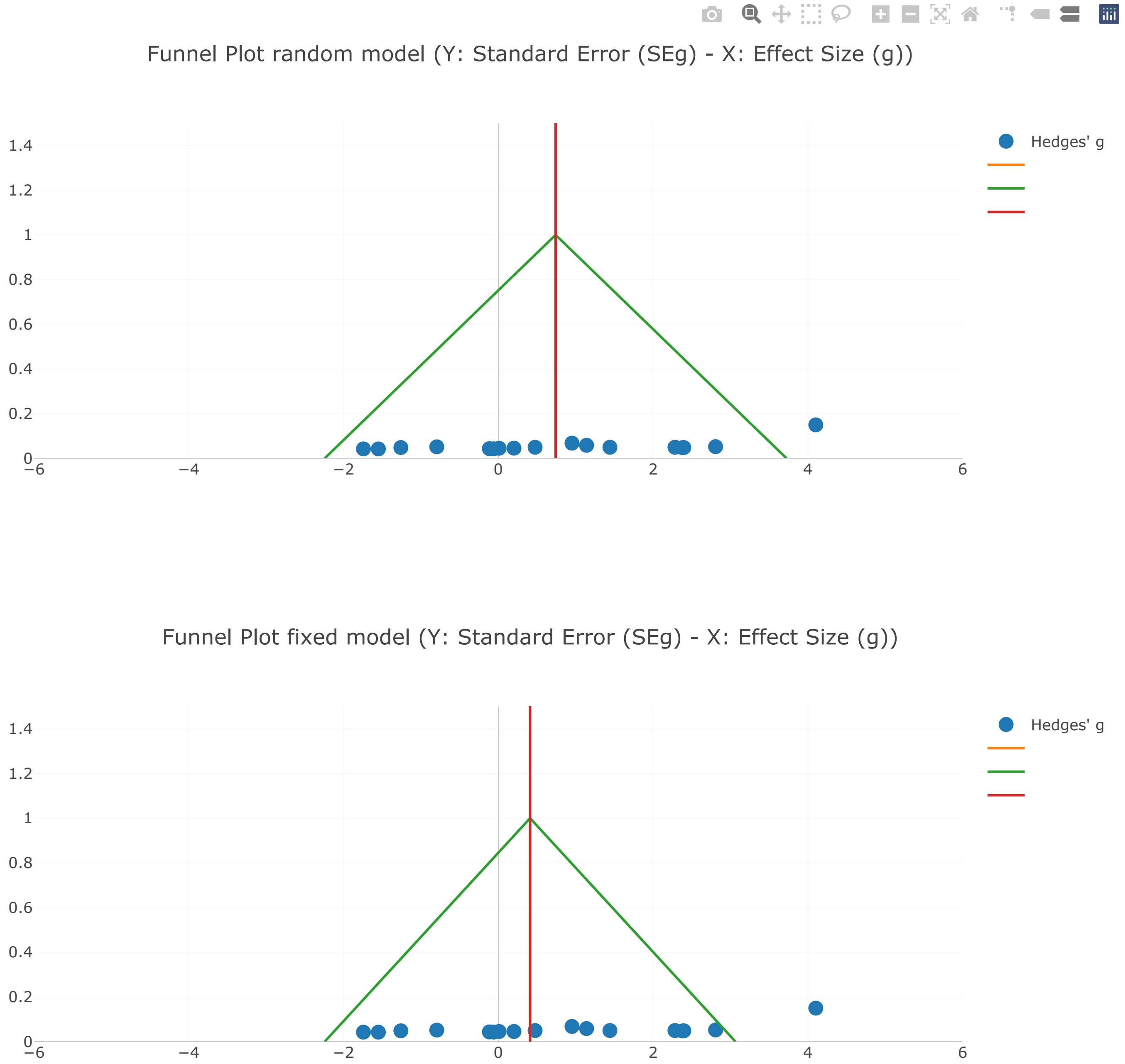

Table.3 Fail-N Safe

Bias of the analysis regarding the file-drawer problem:

Fail-N Safe, the number of studies (or samples) with a null effect (g = 0)
needed to bring the calculated significance level of the pooled effect (p value < 0.0001) near the critical significance level (p value = 0.05),
is calculated as follows:

References:
* Rosenberg, M. S. (2005). The file-drawer problem revisited: a general weighted method for calculating fail-safe numbers in meta-analysis. Evolution, 59(2), 464-468.
* Rosenthal, R. (1979). The file drawer problem and tolerance for null results. Psychological bulletin, 86(3), 638.

	Rosenthal (1979) $t_c(\alpha = 0.05, df = 17) = 1.74$	Rosenberg (2005) $Z_c(\alpha = 0.05) = 1.645$
**Fail-N Safe**	12006.9	6567.66

Table.4 Results of Meta regression

OLS Regression Results

**Dep. Variable:**	y	**R-squared:**	0.000
**Model:**	OLS	**Adj. R-squared:**	0.000
**Method:**	Least Squares	**F-statistic:**	nan
**Date:**	Wed, 31 Mar 2021	**Prob (F-statistic):**	nan
**Time:**	10:39:33	**Log-Likelihood:**	-32.228
**No. Observations:**	17	**AIC:**	66.46
**Df Residuals:**	16	**BIC:**	67.29
**Df Model:**	0		
**Covariance Type:**	nonrobust		

	**coef**	**std err**	**t**	**P>|t|**	**[0.025**	**0.975]**
**const**	0.7447	0.403	1.849	0.083	-0.109	1.598
**Moderator**	0	0	nan	nan	0	0

**Omnibus:**	0.546	**Durbin-Watson:**	0.087
**Prob(Omnibus):**	0.761	**Jarque-Bera (JB):**	0.619
**Skew:**	0.261	**Prob(JB):**	0.734
**Kurtosis:**	2.225	**Cond. No.**	inf

Warnings:
[1] Standard Errors assume that the covariance matrix of the errors is correctly specified.
[2] The smallest eigenvalue is 0. This might indicate that there are
strong multicollinearity problems or that the design matrix is singular.

Table.5 Results of Subgroup Analysis - fixed and random effect models

fixed model

	subgroup analysis
**0**	Oops! There should be at least 2 subgroups for running this analysis!

random model

	subgroup analysis
0	Oops! There should be at least 2 subgroups for running this analysis!

Results of ANOVA for subgroups

F value = 0, p value = 0

Figure.3 Intervals of effect sizes for subgroups - fixed and random effect models

fixed model

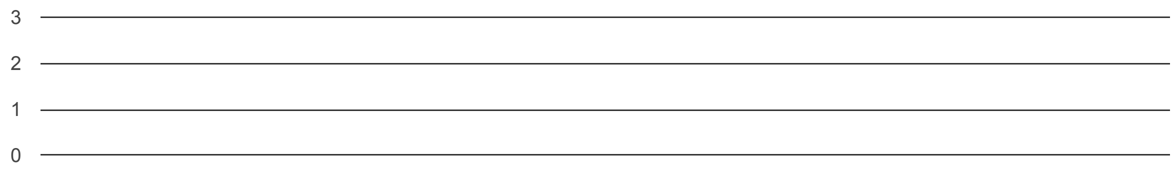

random model

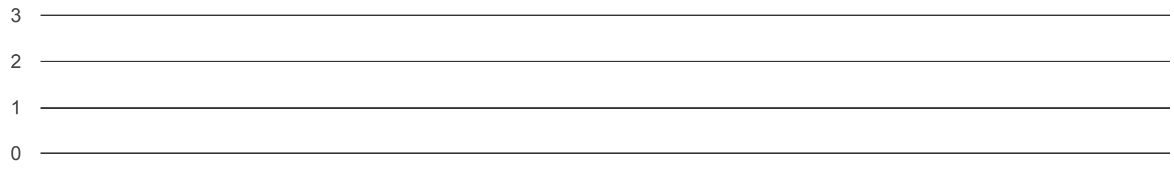

Meta-analysis of pre-existing health conditions in survivors and non-survivors of COVID-19

Table.1 summary of studies

	Study	N1	Mean1	Sd1	N2	Mean2	Sd2	Moderator	subgroup	Cohen's d	CorrectionFactor	Hedges'g (SMD)	SEg	95%CI-Lower	95%CI-Upper	weight(%)-fixed model	weight(%)-random model %
1	Age	5448	46.59	9.06	3927	71.49	6.25	0.0	NAN	3.110953	0.999920	3.110704	0.030890	3.050159	3.171249	3.953468	6.265274
2	Hypertension	9835	16.94	15.67	4245	53.91	16.91	0.0	NAN	2.302867	0.999947	2.302744	0.022924	2.257813	2.347676	7.178531	6.269553
3	Cerebrovascular	3489	3.49	5.85	683	17.30	7.79	0.0	NAN	2.224231	0.999820	2.223831	0.048403	2.128962	2.318700	1.610222	6.251453
4	Diabetes	9877	8.68	7.73	4546	27.71	11.36	0.0	NAN	2.106749	0.999948	2.106640	0.021795	2.063921	2.149358	7.941396	6.270057
5	Comorbidity	7029	34.07	24.14	4444	81.33	23.82	0.0	NAN	1.967809	0.999935	1.967680	0.023151	1.922304	2.013056	7.038445	6.269448
6	Cardiovascular	9557	9.08	8.97	4632	30.27	20.21	0.0	NAN	1.547404	0.999947	1.547322	0.020121	1.507885	1.586759	9.317898	6.270758
7	COPD	7626	4.40	8.29	4299	13.74	7.57	0.0	NAN	1.161996	0.999937	1.161923	0.020501	1.121741	1.202106	8.975512	6.270604
8	Renal	7637	1.94	5.10	3914	10.33	10.97	0.0	NAN	1.101937	0.999935	1.101866	0.020951	1.060802	1.142929	8.594454	6.270418
9	Male	13863	52.90	17.65	4646	66.00	13.47	0.0	NAN	0.784454	0.999959	0.784422	0.017435	0.750250	0.818594	12.410469	6.271766
10	BMI	314	24.14	1.43	69	25.53	3.31	0.0	NAN	0.729024	0.998030	0.727588	0.135274	0.462451	0.992724	0.206155	6.096910
11	Time to Hospital	2109	5.66	3.67	3347	8.00	3.46	0.0	NAN	0.660524	0.999862	0.660433	0.028508	0.604558	0.716308	4.641900	6.266686
12	Liver	3927	4.12	6.47	3856	8.16	8.99	0.0	NAN	0.516580	0.999904	0.516531	0.023044	0.471364	0.561697	7.104075	6.269498
13	Cancer	7156	5.05	15.04	4446	13.40	23.39	0.0	NAN	0.446861	0.999935	0.446832	0.019319	0.408967	0.484697	10.107772	6.271074
14	Other	6465	12.44	19.29	4097	22.65	29.95	0.0	NAN	0.425527	0.999929	0.425497	0.020181	0.385942	0.465052	9.262649	6.270734
15	Smoking history	7230	8.18	7.59	363	9.15	4.94	0.0	NAN	0.129593	0.999901	0.129580	0.053793	0.024146	0.235014	1.303687	6.245987
16	Current drinker	1505	15.60	17.52	101	5.00	7.07	0.0	NAN	-0.621458	0.999532	-0.621167	0.103323	-0.823681	-0.418653	0.353367	6.169781

Figure.1 Forestplot - fixed and random effect models

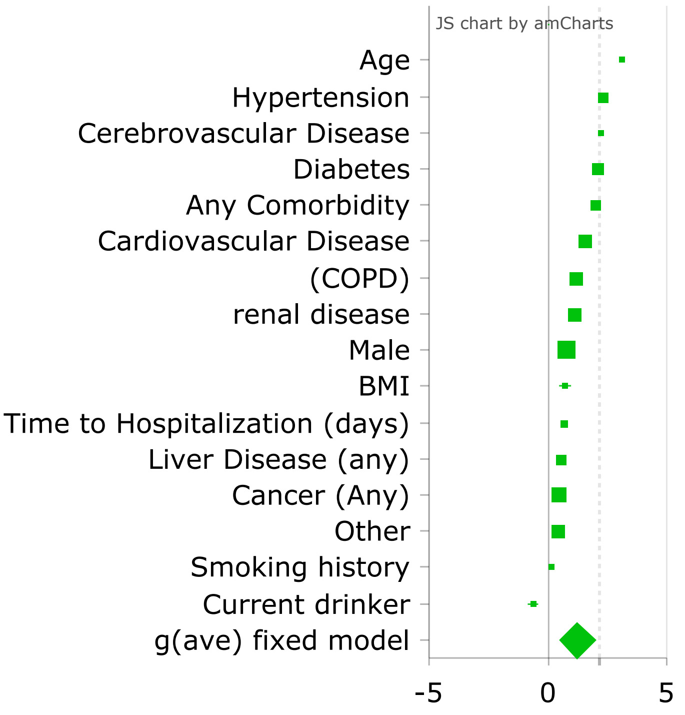

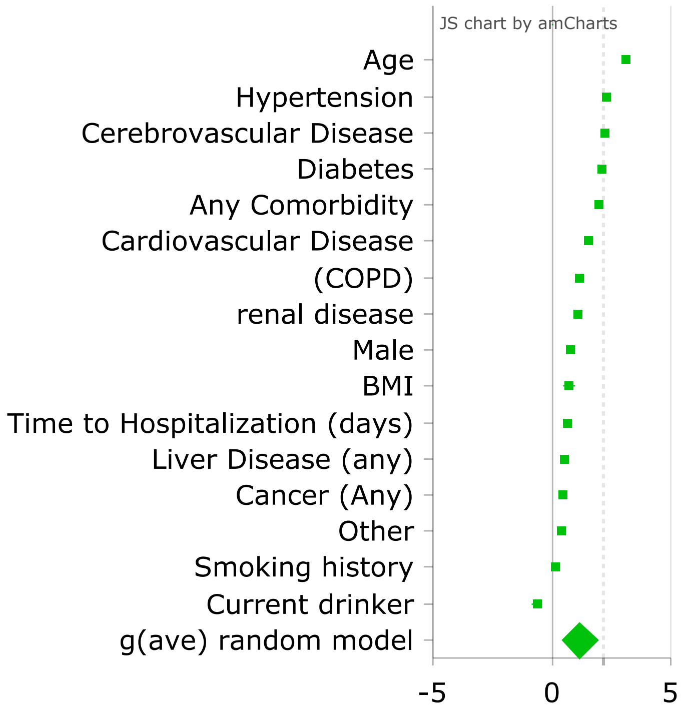

Figure.2 Funnel Plot - fixed and random effect models

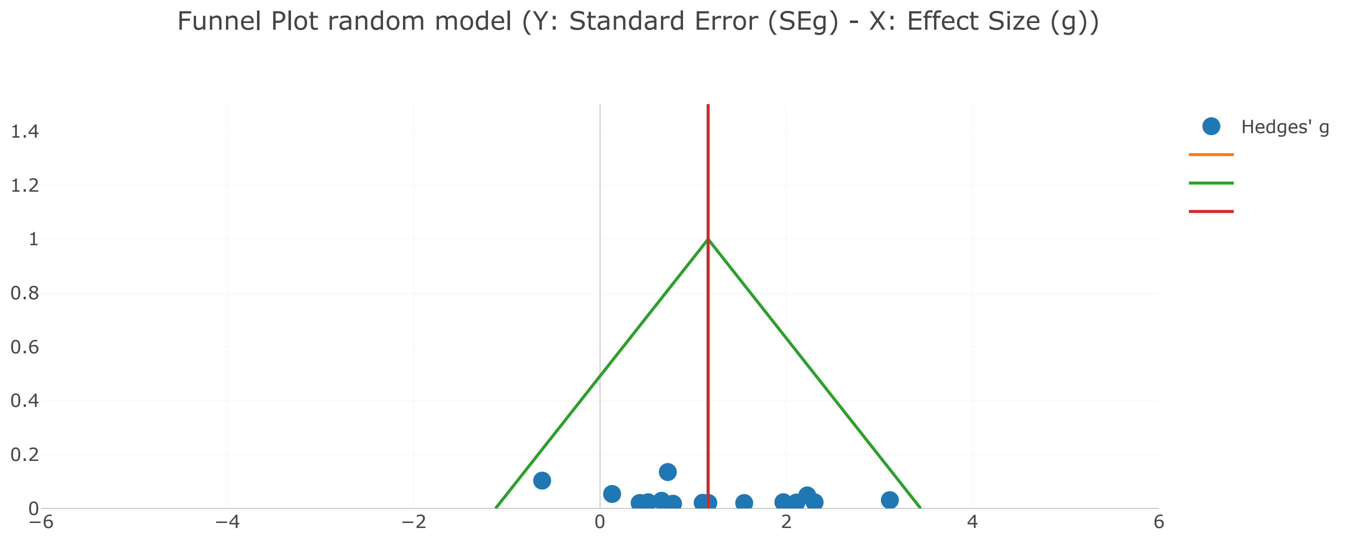

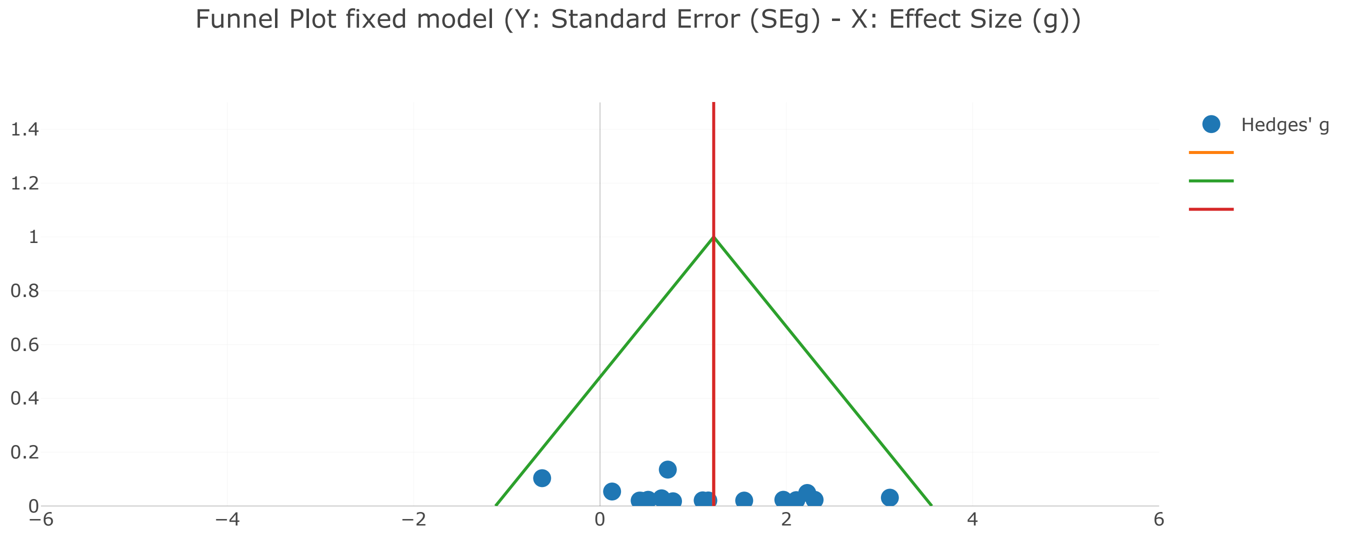

Table.3 Fail-N Safe

Bias of the analysis regarding the file-drawer problem:

Fail-N Safe, the number of studies (or samples) with a null effect (g = 0)
needed to bring the calculated significance level of the pooled effect (p value < 0.0001) near the critical significance level (p value = 0.05),
is calculated as follows:

References:
* Rosenberg, M. S. (2005). The file-drawer problem revisited: a general weighted method for calculating fail-safe numbers in meta-analysis. Evolution, 59(2), 464-468.
* Rosenthal, R. (1979). The file drawer problem and tolerance for null results. Psychological bulletin, 86(3), 638.

	Rosenthal (1979) $t_c(\alpha = 0.05, df = 16) = 1.746$	Rosenberg (2005) $Z_c(\alpha = 0.05) = 1.645$
**Fail-N Safe**	208450.49	208063.16

Table.4 Results of Meta regression

OLS Regression Results

**Dep. Variable:**	y	**R-squared:**	0.000
**Model:**	OLS	**Adj. R-squared:**	0.000
**Method:**	Least Squares	**F-statistic:**	nan
**Date:**	Wed, 31 Mar 2021	**Prob (F-statistic):**	nan
**Time:**	11:10:41	**Log-Likelihood:**	-21.759
**No. Observations:**	16	**AIC:**	45.52
**Df Residuals:**	15	**BIC:**	46.29
**Df Model:**	0		
**Covariance Type:**	nonrobust		

	coef	std err	t	P>|t|	[0.025	0.975]
**const**	1.1620	0.243	4.774	0.000	0.643	1.681
**Moderator**	0	0	nan	nan	0	0

**Omnibus:**	0.290	**Durbin-Watson:**	0.127
**Prob(Omnibus):**	0.865	**Jarque-Bera (JB):**	0.391
**Skew:**	0.257	**Prob(JB):**	0.822
**Kurtosis:**	2.431	**Cond. No.**	inf

Warnings:
[1] Standard Errors assume that the covariance matrix of the errors is correctly specified.
[2] The smallest eigenvalue is 0. This might indicate that there are
strong multicollinearity problems or that the design matrix is singular.

Table.5 Results of Subgroup Analysis - fixed and random effect models

fixed model

	subgroup analysis
**0**	Oops! There should be at least 2 subgroups for running this analysis!

random model

	subgroup analysis
**0**	Oops! There should be at least 2 subgroups for running this analysis!

Results of ANOVA for subgroups

F value = 0, p value = 0

Figure.3 Intervals of effect sizes for subgroups - fixed and random effect models

fixed model

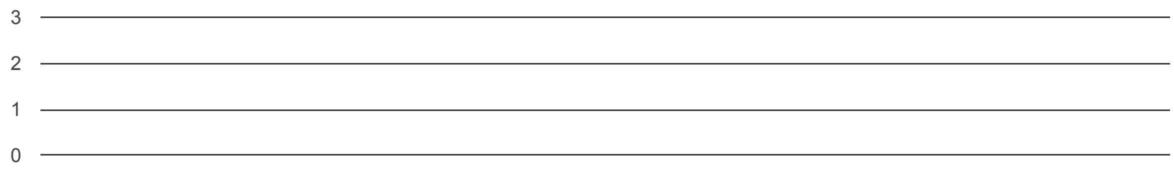

random model

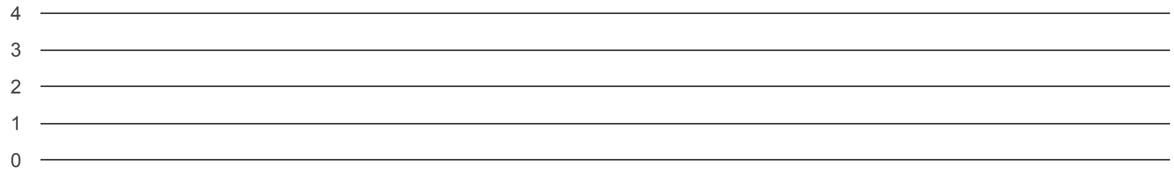

Meta-analysis of complications in survivors and non-survivors of COVID-19

Table.1 summary of studies

	Study	N1	Mean1	Sd1	N2	Mean2	Sd2	Moderator	subgroup	Cohen's d	CorrectionFactor	Hedges'g (SMD)	SEg	95%CI-Lower	95%CI-Upper	weight(%)-fixed model	weight(%)-random model %
1	Age	5448	46.59	9.06	3927	71.49	6.25	0.0	NAN	3.110953	0.999920	3.110704	0.030890	3.050159	3.171249	18.011447	10.028482
2	Heart failure	1780	0.98	3.06	368	47.80	13.74	0.0	NAN	7.398459	0.999650	7.395873	0.126528	7.147878	7.643869	1.073536	9.924302
3	Septic shock / shock	4112	0.64	1.33	455	47.22	32.61	0.0	NAN	4.495674	0.999836	4.494935	0.068207	4.361249	4.628621	3.694321	10.002691
4	Acidosis	357	1.40	3.37	167	21.82	11.05	0.0	NAN	2.992127	0.998563	2.987826	0.131461	2.730161	3.245490	0.994478	9.915595
5	Respiratory failure or ARDS	2546	16.29	26.50	3767	82.12	21.18	0.0	NAN	2.804749	0.999881	2.804416	0.035791	2.734266	2.874566	13.416828	10.026198
6	Acute cardiac injury	1655	4.57	6.78	3824	46.09	25.85	0.0	NAN	1.894513	0.999863	1.894253	0.034539	1.826556	1.961950	14.406906	10.026813
7	Coagulopathy	913	0.85	2.09	373	20.84	20.42	0.0	NAN	1.795815	0.999416	1.794766	0.070882	1.655838	1.933695	3.420749	10.000103
8	Acute kidney injury (AKI)	3202	4.16	9.75	3734	24.42	14.23	0.0	NAN	1.638465	0.999892	1.638288	0.027811	1.583778	1.692798	22.220222	10.029746
9	Secondary infection/ Bacterial infection	1537	7.81	15.55	3456	43.53	31.14	0.0	NAN	1.308077	0.999850	1.307881	0.033331	1.242551	1.373210	15.469820	10.027386
10	Liver dysfunction	2527	22.25	26.16	510	24.86	24.20	0.0	NAN	0.101000	0.999753	0.100975	0.048549	0.005818	0.196131	7.291693	10.018683

Figure.1 Forestplot - fixed and random effect models

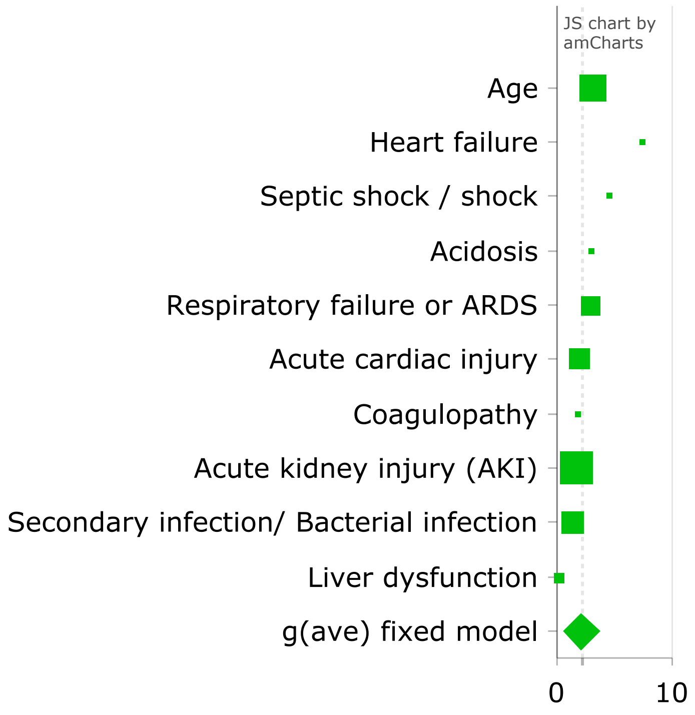

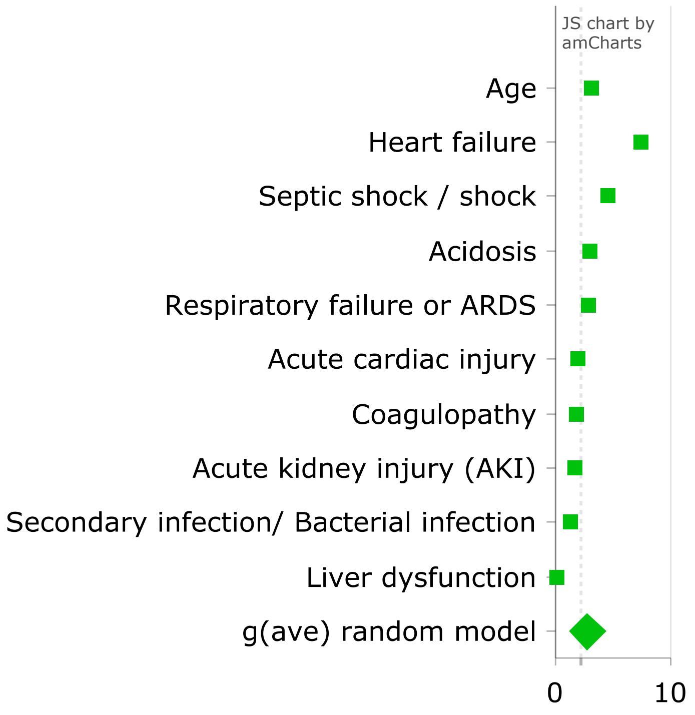

Figure.2 Funnel Plot - fixed and random effect models

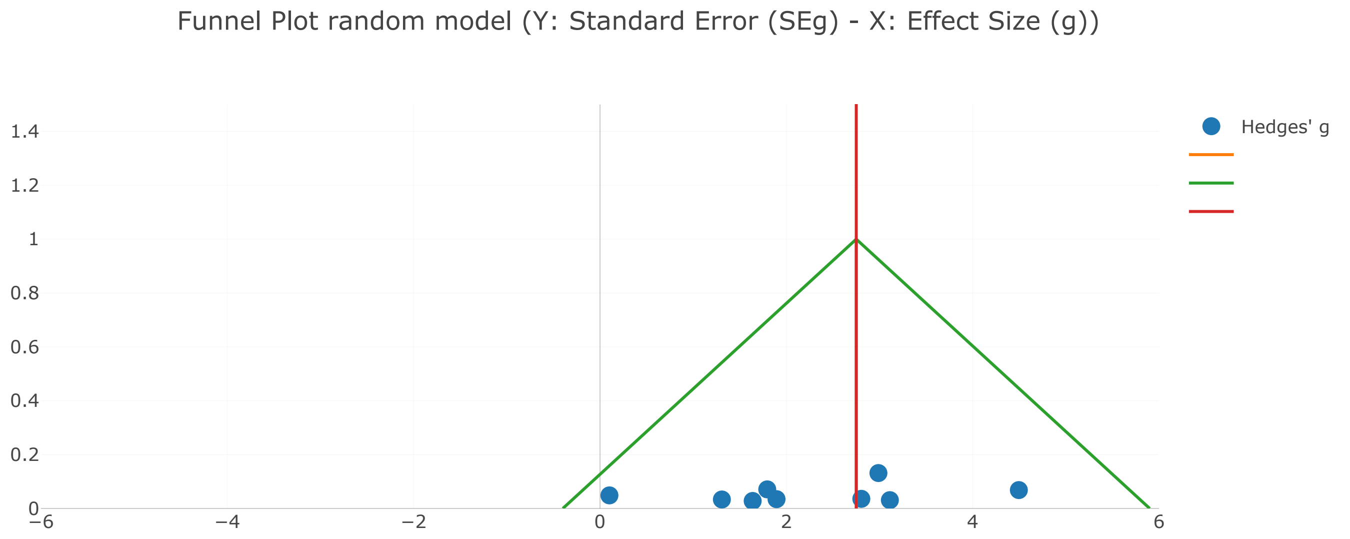

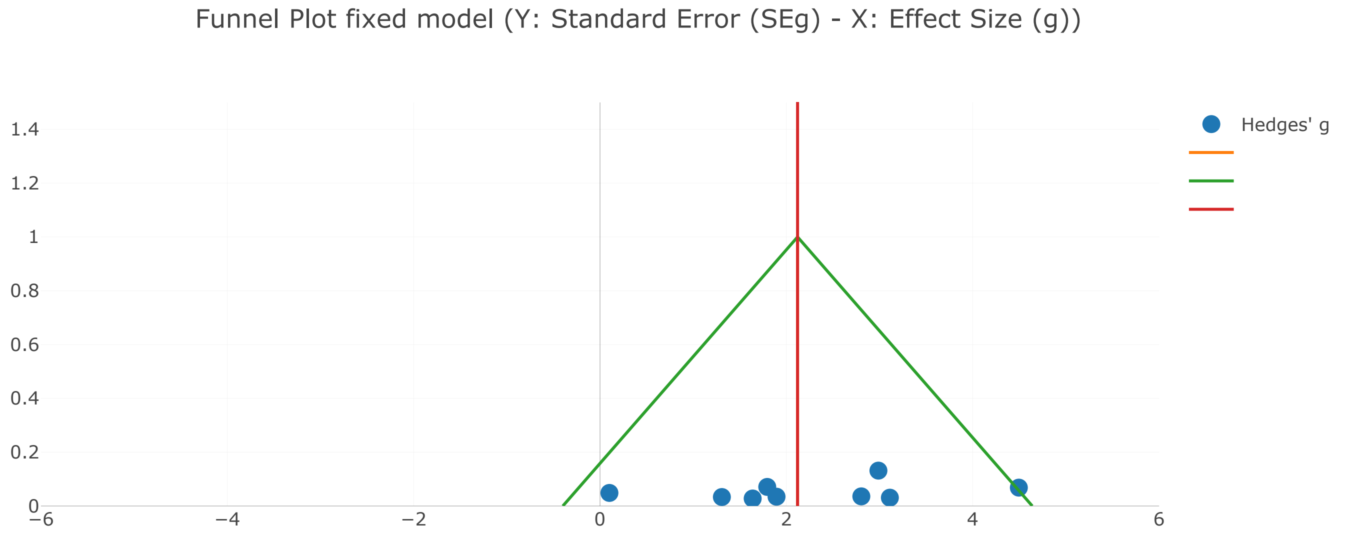

Table.3 Fail-N Safe

Bias of the analysis regarding the file-drawer problem:

Fail-N Safe, the number of studies (or samples) with a null effect (g = 0)
needed to bring the calculated significance level of the pooled effect (p value < 0.0001) near the critical significance level (p value = 0.05),
is calculated as follows:

References:
* Rosenberg, M. S. (2005). The file-drawer problem revisited: a general weighted method for calculating fail-safe numbers in meta-analysis. Evolution, 59(2), 464-468.
* Rosenthal, R. (1979). The file drawer problem and tolerance for null results. Psychological bulletin, 86(3), 638.

	Rosenthal (1979) $t_c(\alpha = 0.05, df = 10) = 1.812$	Rosenberg (2005) $Z_c(\alpha = 0.05) = 1.645$
**Fail-N Safe**	94804.91	79575.22

Table.4 Results of Meta regression

OLS Regression Results

**Dep. Variable:**	y	**R-squared:**	0.000
**Model:**	OLS	**Adj. R-squared:**	0.000
**Method:**	Least Squares	**F-statistic:**	nan
**Date:**	Wed, 31 Mar 2021	**Prob (F-statistic):**	nan
**Time:**	11:26:24	**Log-Likelihood:**	-20.703
**No. Observations:**	10	**AIC:**	43.41
**Df Residuals:**	9	**BIC:**	43.71
**Df Model:**	0		
**Covariance Type:**	nonrobust		

	coef	std err	t	P>|t|	[0.025	0.975]
**const**	2.7530	0.639	4.306	0.002	1.307	4.199
**Moderator**	0	0	nan	nan	0	0

**Omnibus:**	6.693	**Durbin-Watson:**	0.856
**Prob(Omnibus):**	0.035	**Jarque-Bera (JB):**	2.539
**Skew:**	1.146	**Prob(JB):**	0.281
**Kurtosis:**	3.919	**Cond. No.**	inf

Warnings:
[1] Standard Errors assume that the covariance matrix of the errors is correctly specified.
[2] The smallest eigenvalue is 0. This might indicate that there are
strong multicollinearity problems or that the design matrix is singular.

Table.5 Results of Subgroup Analysis - fixed and random effect models

fixed model

	subgroup analysis
**0**	Oops! There should be at least 2 subgroups for running this analysis!

random model

	subgroup analysis
**0**	Oops! There should be at least 2 subgroups for running this analysis!

Results of ANOVA for subgroups

F value = 0, p value = 0

Figure.3 Intervals of effect sizes for subgroups - fixed and random effect models

fixed model

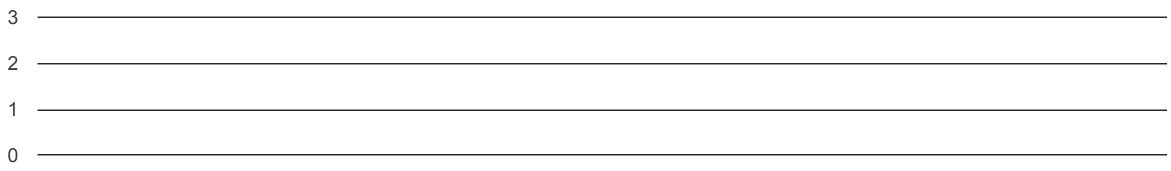

random model

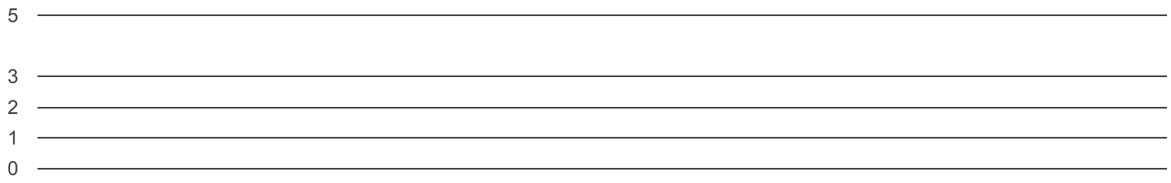

Supplement: Supplementary file 3 — Supplementary Information 3. [file 41598_2023_29364_MOESM3_ESM.pdf]
